# Chemical and molecular characterization of metabolites from *Flavobacterium sp*.

**DOI:** 10.1371/journal.pone.0205817

**Published:** 2018-10-17

**Authors:** Vildan Enisoglu-Atalay, Belkis Atasever-Arslan, Bugra Yaman, Rumeysa Cebecioglu, Aykut Kul, Selma Ozilhan, Fatih Ozen, Tunc Catal

**Affiliations:** 1 Istanbul Research Application and Inovation Center (PROMER), Uskudar University, Uskudar, Istanbul, Turkey; 2 Department of Bioengineering, Uskudar University, Uskudar, Istanbul, Turkey; 3 Department of Molecular Biology and Genetics, Uskudar University, Uskudar, Istanbul, Turkey; 4 Personalized Medicine Application and Research Center (KIMER), Uskudar University, Uskudar, Istanbul, Turkey; Institute of Medical Research and Medicinal Plant Studies, CAMEROON

## Abstract

In this study, a *Flavobacterium sp*. is isolated from natural spring, and identified using molecular techniques. Extracellular and intracellular secondary metabolites are identified using solid phase microextraction gas chromatography-mass spectrometry and ultra performance liquid chromatography. Cytotoxic activity of the extracellular compounds produced by the *Flavobacterium* sp. and quercetin as the standard are measured using ECV304 human endothelial cells in vitro. Our results show that *Flavobacterim* sp. isolate has the highest percentage of similarity with *Flavobacterium cheonhonense* strain ARSA-15 (99%). Quercetin is detected as the major extracellular compound produced by the *Flavobacterium* sp. Methanol extract of *Flavobacterium* sp. resulted in a higher cell viability results when compared to DMSO extracts. Computational chemistry approach was used and it has been found that polar solvent (methanol) contributed to higher antioxidant activity. In conclusion, *Flavobacterium* sp. can be used to produce quercetin for industrial purposes.

## Introduction

In recent years, many researches have been focused on *Flavobacterium* sp. due to their biologically interesting features. *Flavobacterium* is a genus of gram-negative, motile or non-motile, aerobic or facultatively anaerobic, rod shaped bacteria, characteristically producing yellow, red, orange or yellow-brown pigmentation. They are found in freshwater or in soil. Several species of *Flavobacterium* have been found to be pathogenic to several organisms such as fish, algae and soil organisms. However, several studies show that *Flavobacterium* genus covers many interesting species to produce important metabolites and end products. For example, *Flavobacterium* can be used extensively in carotenoid production such as zeaxanthin. Besides zeaxanthin, several strains of *Flavobacterium* have been identified for production of β-carotene which can be used commercially as food dyes and food supplements [1]. Especially, due to its therapeutic properties on antioxidant activities and chronic diseases, it is widely used in health field for pharmaceutical purposes [1]. Species of *Flavobacterium* have been found to produce a variety of compounds to oxidize a range of aromatic hydrocarbons. A variety of enzymes involved in the degradation of agar, alginate, chitin, pectin, xylan, keratin, laminarin have been found to be produced by *Flavobacterium* sp. Polysaccharide-degrading enzymes which can degrade the cell wall of various pathogens and protease enzymes, isoamylase, the recombinant β-glucosidase can be also produced by *Flavobacterium sp* [2,3]. Oxidation of ethane, propane and butane is also carried out by *Flavobacterium sp*. *Flavobacterium* sp. have been reported to secrete antibiotic constituents. Several antifungal compounds such as deacetoxycephalosprin C and a mixture of 7 substituted cephalosporins are secreted by *Flavobacterium* [4]. Flavocristamides A & B were isolated from a marine bacterium *Flavobacterium sp*. which represent inhibitory activity against DNA polymerase. The isolation and identification of chitinovorins A, B and C were observed from the culture broth of *Flavobacterium chitinovorum*, which belong to the class of β-lactam antibiotics. Chitinovorin D was later isolated from a *Flavobacterium sp*. PB-5246 which is more strongly basic antibiotic [5]. In this respect, isolation and identification of *Flavobacterium* and understanding metabolites produced by the bacteria is important to open new research gates. So far, there is no information about isolated *Flavobacterium* secreting quercetin and novel important metabolites by *Flavobacterium* sp. has not been reported, yet.

In this study, quercetin production by a *Flavobacterium* sp. isolate was discovered for the first time in literature. 16S rRNA gene sequencing has been applied to identify the species of the bacterial isolate. Secondary metabolites were analyzed using solid phase microextraction-gas chromatography (SPME-GC/MS) and ultra-pressure liquid chromatography (UPLC). Cytotoxic activities of extracellular quercetin produced by the bacteria was measured using the MTT (3-(4,5-dimethylthiazol-2-yl)-2,5-diphenyltetrazolium bromide) tetrazolium reduction) assay. Computational chemistry approach was used in this study calculating the following parameters; the highest occupied molecular orbital (HOMO)-lowest unoccupied molecular orbital (LUMO) energy values of frontier orbitals, and their distribution, and ionization potentials (IPs) for quercetin. The HOMO-LUMO energies were determined by using TD-DFT/M06-2X//6-311++G(d,p) levels in examined phases.

## Methods

### 2.1. Isolation of *Flavobacterium* sp. and culture condition

A water sample was obtained from Resadiye region (41°04'35.6"N 29°15'02.4"E, Istanbul, Turkey) from natural spring water and transferred to laboratory, and no specific permissions were required for these locations/activities. Bristol medium was used in culturing of microorganisms. The following stock solutions were prepared: NaNO_3_ solution (25 g/L), CaCl_2_.2H_2_O solution (2.5 g/L), MgSO_4_.7H_2_O solution (7.5 g/L), K_2_HPO_4_ solution (7.5 g/L), KH_2_PO_4_ solution (17.5 g/L) and NaCl solution (2.5 g/L). 10 mL of each stock solution was added into 940 mL of distilled water, and finally 1 g/L of peptone was added. For agar plates, 1.5% of agar was added to the solution. The Bristol medium was autoclaved at 121°C for 15 min before inoculations. Agar plates were inoculated with microbial culture using loop under aseptic conditions. Cells were grown in 100 mL shake flask containing 60 mL of Bristol Medium for 7 days inside the incubator (Thermo Scientific, model no.: 4339, USA) under 24h of illumination at 28°C.

### 2.2. DNA extraction

*Flavobacterium* sp. was grown in Bristol Medium for 7 days. 1 mL sample (n = 2) was taken for DNA extraction. DNA was extracted using a commercial DNA isolation kit (Zymo Research Company, Fungal/Bacterial DNA miniprep, Catalog No: D6006, Lot No: ZRC180906). Extracted DNA samples were stored in a deep-freezer at -20°C.

### 2.3. PCR analysis and DNA sequencing

PCR consisted of [1 μL each primer and 1 μL dNTP, 0.5 μL Taq polymerase, 1.5 μL Mg, 2 μL Buffer, 8 μL H_2_O (x3), and 5 μL DNA served as a template in 20 μL total PCR volume. Reactions were performed in a Thermal Cycler (BIO-RAD T100, Singapore) (PCR protocol: Lid 105°C, Volume: 20 μL, 1: 95°C 1 min, 2 : 95°C 30 sec, 3 : 55°C 30 sec, 4 : 72°C 1 min, 5 : GOTO step 2, 24x, 6 : 72°C 5 min, 7 : 4°C). The following primers obtained from Medsantek Company (Istanbul, Turkey) were used in PCR: forward primer; 63f (59-CAGGCC TAA CAC ATG CAA GTC-39), reverse primer; 1387r (59-GGG CGG WGT GTA CAA GGC-39). PCR products were stored at -20°C. PCR products were sequenced using Genetic Analyzer 3500 xL (Medsantek, Istanbul, Turkey), and results were evaluated using Chromas Software (version 2.6.2).

### 2.4. Methanol extraction of *Flavobacterium* sp.

Culture broth containing bacteria were taken from shake flasks, and centrifuged at 4°C at 4,500 rpm for 20 min. 0.1% of butylated hydroxytoluene (BHT) was added to the supernatant, and was stored at 4°C for GC-MS analysis. The sample held in the light was measured and 0.05 g was weighed. Absolute methanol containing 0.1% of BHT was added to the bacterial pellet, and vortexed for 30 sec. The mixture was placed into an ultrasonicator (Bandelin) and ultrasonicated for 30 sec. Ultrasonicated sample was centrifuged at 4°C at 4,500 rpm for 20 min. The supernatant was stored at 4°C until GC-MS analysis.

### 2.5. SPME-GC-MS analysis

Extracts were analyzed by The Scientific and Technological Research Council of Turkey, Marmara Research Center (TÜBİTAK-MAM, Gebze, Turkey) using SPME-GC-MS system (Analysis no. G345, Xcalibur Thermo Scientific, USA). Qualitative analysis results of extracts were used to determine specific quantitative assay for active metabolites. Peak areas of the substances were calculated by Xcalibur, and relative amounts of the substances were calculated on the basis of peak-area ratios.

### 2.6. Biochemical analysis

Alpha and beta acids in methanol extract of *Flavobacterium* sp. biomass were measured spectrophotometrically according to a previous report [6]. 100 mL alkaline methanol was prepared with 0.2 mL 6.0 N NaOH. The sample mixed into 50 mL toluene and shaked for 30 min. 1.5 mL of solution was pipetted into plastic vessels and centrifuged at 5.000 rpm for 5 min. 0.25 mL of the solution was diluted with 4.75 mL of methanol (dilution A). And, 0.25 mL of toluene was added into 4.75 mL of absolute methanol as a blank. The samples were measured using a UV spectrophotometer (Thermo Multiscan 200–1000 nm, USA). 0.1 mL of blank pipetted with 2.0 mL of alkaline methanol, shaked and calibrated. 0.1 ml of solution added into 2.0 ml alkaline methanol (dilution B) and absorbances were read at 275 nm, 325 nm, 355 nm. The percentages of alpha and beta acids were calculated using the following equations:

Dilutionfactor,d=(volumedilutionAxvolumedilutionB)/(500xaliqextractAxaliqdilA)

%alphaacids=dx(‑51.56A355+73.79A325‑19.07A275)

%betaacids=dx(55.57A355‑47.59A325+5.10A275)

### 2.7. UPLC analysis

Chromatographic separation was achieved using a Waters Acquity UPLC System consisting of a sample manager, a column heater/cooler, a binary solvent manager, and 10.0 μL injection loop. The chromatographic separation was achieved using an ACQUITY UPLC HSS C18 (1.8 μm, 2.1 x 150 mm) column. The acquity autosampler temperature was set at 10°C and the injection volume was 5.0 μL. The column was maintained at 50°C. Mobile phase A consisted of 5.0 mM ammonium formate buffer (pH = 3.0) while mobile phase B consisted of acetonitrile with 0.1% formic acid.

The flow rate of the mobile phase under gradient condition was kept at 0.5 mL/min. The gradient consisted of linear gradient from 4% B to 50% B (0–2 min), back to 4% B (2–3 min) and held constant at 95% B (3–4 min). The total run time was 4.0 min. The detection of quercetin was performed using a Waters Xevo TQD (triple quadrupole) tandem mass spectrometer (Waters Corp., Milford, MA, USA) with an electrospray ionization source in negative ionization mode. Quantitation was performed using multiple reactions monitoring (MRM) mode to study parent → product ion (m/z) transitions quercetin (301.0 /150.9). The MS/MS instrument parameters were optimized, including capillary voltage 3.0 kV, nitrogen gas temperature 450°C, source temperature 150°C, collision gas flow 0.22 mL/min, nitrogen gas flow 20 L/h, and desolvation gas flow 1,000 L/h. Preparation of Reagents and Standards: Calibration standards for Quercetin: 6.25, 12.5, 25.0, 50.0 100.0, and 250.0 ng/mL and were prepared by spiking methanol.

### 2.8. Cytotoxicity assay

The cytotoxic effects of *Flavobacterium sp*. *extracellular* extract and quercetin against Ishikawa cells and ECV304 human endothelial cells were measured *via* MTT (3-(4,5-dimethylthiazol-2yl)-2,5-diphenyl tetrazolium bromide) (Sigma, M-5655) assay [7]. Cell line was obtained from American Type Culture Collection (ATCC, USA). 1×10^5^ cells/mL were suspended in the medium supplemented with 10% of fetal bovine serum, 1% of penicillin-streptomycin, 1% of L-glutamine solution and 0.1% of MEM nonessential amino acids solution (100×) at 37°C in a humidified atmosphere containing 5% CO_2_.

Stock solutions of the *Flavobacterium* sp. extract were prepared as a final concentration of 10 mg/mL using dimethyl sulfoxide (DMSO). Stock solution of the quercetin (Sigma-Aldrich, USA) (10 mg/mL concentration) was prepared using DMSO. Five different concentrations for both were prepared by serial dilutions of the stock solution: 50, 10, 5, 0 μg/mL. Ten μL dilution of the *Flavobacterium* extract and quercetin dispensed into 96-well plates, respectively, and 90 μL of the cells was added into each well. Final concentrations of 50, 10, 5, 0 μg/mL were used in the experiments (n = 6, for each). For the control wells, 10 μL medium was used instead of *Flavobacteriım* extract. After 48 hours of incubation, 10 μL of MTT (5 mg/mL) solutions in phosphate buffer saline (PBS) were added into each well and incubated them for 3 h at 37°C. After the incubation supernatants were from all wells and 100 μL of SDS (pH 5.5) containing isopropyl alcohol was added in order to dissolve the formazan crystals formed by reduction of MTT in living cells. Then, microplates were left in the dark room for 10 min. Optical density of each well was measured with 570 nm on a microplate reader (Thermo Multiskan 200-1000nm, USA). Cytotoxic index (CI) was calculated with the formula:

Cytotoxicindex=1−[OD(treatedwells)/OD(controlwells)]×100%.

### 2.9. Computational methods

Quercetin molecular geometry optimizations were calculated by DFT/M06-2X method with 6-31Gd basis set. The Single Point Energies of the ground state, anion, cation and radical structures and TD-SCF calculations which used to determine HOMO-LUMO energies were performed at the DFT/M06-2X/6-311++Gdp level of theory. All studied structures were optimized in gas, DMSO, methanol and ethanol phases. For the solvent optimization IEF-PCM method was used. All computational calculations were carried out through the GaussView5 [8] molecular visualization program and Gaussian09 program package [9].

## Results

### 3.1. Molecular identification results

**Fig 1:**
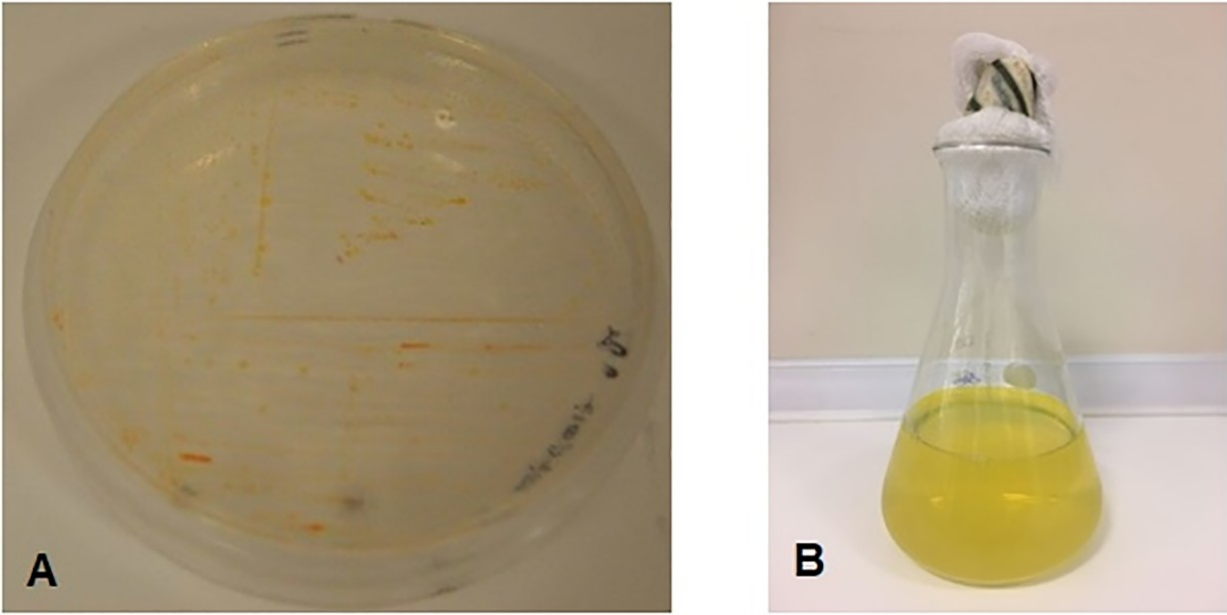
**Image of *Flavobacterium sp*. grown on agar plate (a), and liquid culture (b)**.

**Fig 2:**
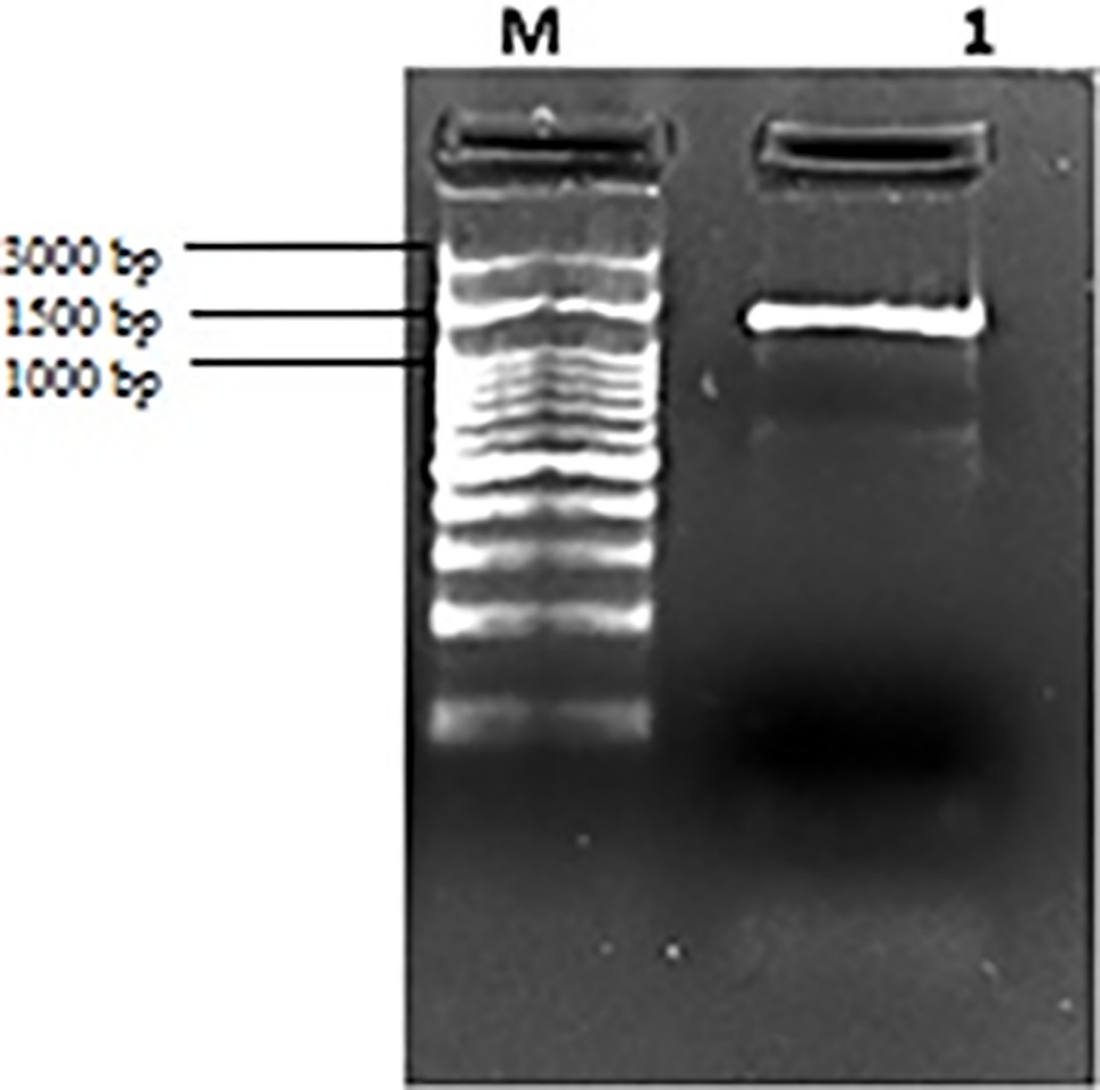
Electrophoresis of single band of 16S rDNA PCR, M: Marker.

The bacteria produced intense yellow colored extracellular substances (Fig 1A and 1B). After DNA isolation and PCR amplification, PCR products were run in gel electrophoresis (Fig 2). Molecular identification results showed that *Flavobacterium* isolate has the highest percentage of similarity with *Flavobacterium cheonhonense* strain ARSA-15, 16S ribosomal RNA gene with a 99% level value (GenBank Acces. No. KY971497). In this respect, it can be suggested that *Flavobacterium* sp. isolate is *Flavobacterium cheonhonense* strain ARSA-15.l.

### 3.2. SPME-GC-MS results

Table 1 shows that SPME-GC-MS analysis of supernatant of *Flavobacterium* sp. containing extracellular substances. The following quinone derivatives were detected in extracellular media; 6,7-dimethoxy-2-methyl-3,4-dihydro[1-D] isoquinolinium ion, 5,6-dihydro-2,4-dimethylbenz[f] isoquinoline, quercetin 7,3',4'-trimethoxy, 3-(2,2-dimethyl propylidene) bicyclo[3.3.1] nonane-2,4-dione. Table 2 shows GC-MS analysis of methanol extract of *Flavobacterium* isolate. Lupulon was detected in methanol extract of *Flavobacterium* sp. biomass.

**Table 1:** SPME-GC-MS analysis of supernatant of *Flavobacterium sp*. SI, direct matching factor, RSI; reverse search matching factor.

RT	Name	Molecular formula	SI	RSI	Area (%)
2.27	Cyclotetrasiloxane, octamethyl-	C_8_H_24_O_4_Si_4_	752	779	2.39
2.66	Silicic acid, diethyl bis(trimethylsilyl) ester	C_10_H_28_O_4_Si_3_	722	776	3.18
3.46	6,7-Dimethoxy-2-methyl-3,4-dihydro[1-D] isoquinolinium ion	C_12_H_17_NO_2_	745	864	2.51
5.31	Cyclotrisiloxane, hexamethyl-	C_6_H_18_O_3_Si_3_	744	872	6.41
6.34	Cyclotrisiloxane, hexamethyl-	C_6_H_18_O_3_Si_3_	777	871	9.79
6.56	Cyclotrisiloxane, hexamethyl-	C_6_H_18_O_3_Si_3_	728	875	2.49
8.0	2-Myristynoyl-glycinamide	C_16_H_28_N_2_O_2_	563	620	2.14
10.28	Cyclohexasiloxane, dodecamethyl-	C_12_H_36_O_6_Si_6_	902	971	4.89
11.37	Nonanal	C_9_H_18_O	792	899	6.63
16.10	5,6-Dihydro-2,4-dimethylbenz[f] isoquinoline	C_15_H_15_N	773	847	5.01
16.99	Cycloheptasiloxane, tetradecamethyl-	C_14_H_42_O_7_Si_7_	761	789	19.60
22.27	Quercetin 7,3',4'-trimethoxy	C_18_H_16_O_10_	586	600	5.10
23.49	Cyclooctasiloxane, hexadecamethyl-	C_16_H_48_O_8_Si_8_	825	847	9.83
25.75	Methoxy, phenyl-, oxime	C_8_H_9_NO_2_	721	836	4.03

RT: Retention time

**Table 2:** SPME-GC-MS analysis of methanol extract of *Flavobacterium sp*. biomass. SI, direct matching factor, RSI; reverse search matching factor.

RT	Name	Molecular formula	SI	RSI	Area (%)
4.75	Cyclopentasiloxane, decamethyl-	C_10_H_30_O_5_Si_5_	809	829	0.51
6.34	Cyclotrisiloxane, hexamethyl-	C_6_H_18_O_3_Si_3_	779	874	1.97
7.24	1,1,3,3,5,5,7,7,9,9,11,11,13,13-tetradecamethylheptasiloxane	C_14_H_42_O_6_Si_7_	672	894	0.59
10.31	Cyclohexasiloxane, dodecamethyl-	C_12_H_36_O_6_Si_6_	909	972	2.78
10.80	Lupulon	C_26_H_38_O_4_	581	607	0.54
16.98	Cycloheptasiloxane, tetradecamethyl-	C_14_H_42_O_7_Si_7_	772	795	4.04
23.48	Cyclooctasiloxane, hexadecamethyl-	C_16_H_48_O_8_Si_8_	821	856	0.91
30.16	Benzene, 1,1'-(3-methyl-1,3-butadienylidene)bis-	C_17_H_16_	949	955	82.46
41.55	Benzoic acid, silver (1+) salt	C_7_H_5_AgO_2_	741	893	0.51
45.80	Methyl 2-{4'-[(E)-2"-Nitroethenyl]phenyloxy}ethyl pentanedioate	C_16_H_19_NO_7_	660	911	1.91

RT: Retention time

**Fig 3:**
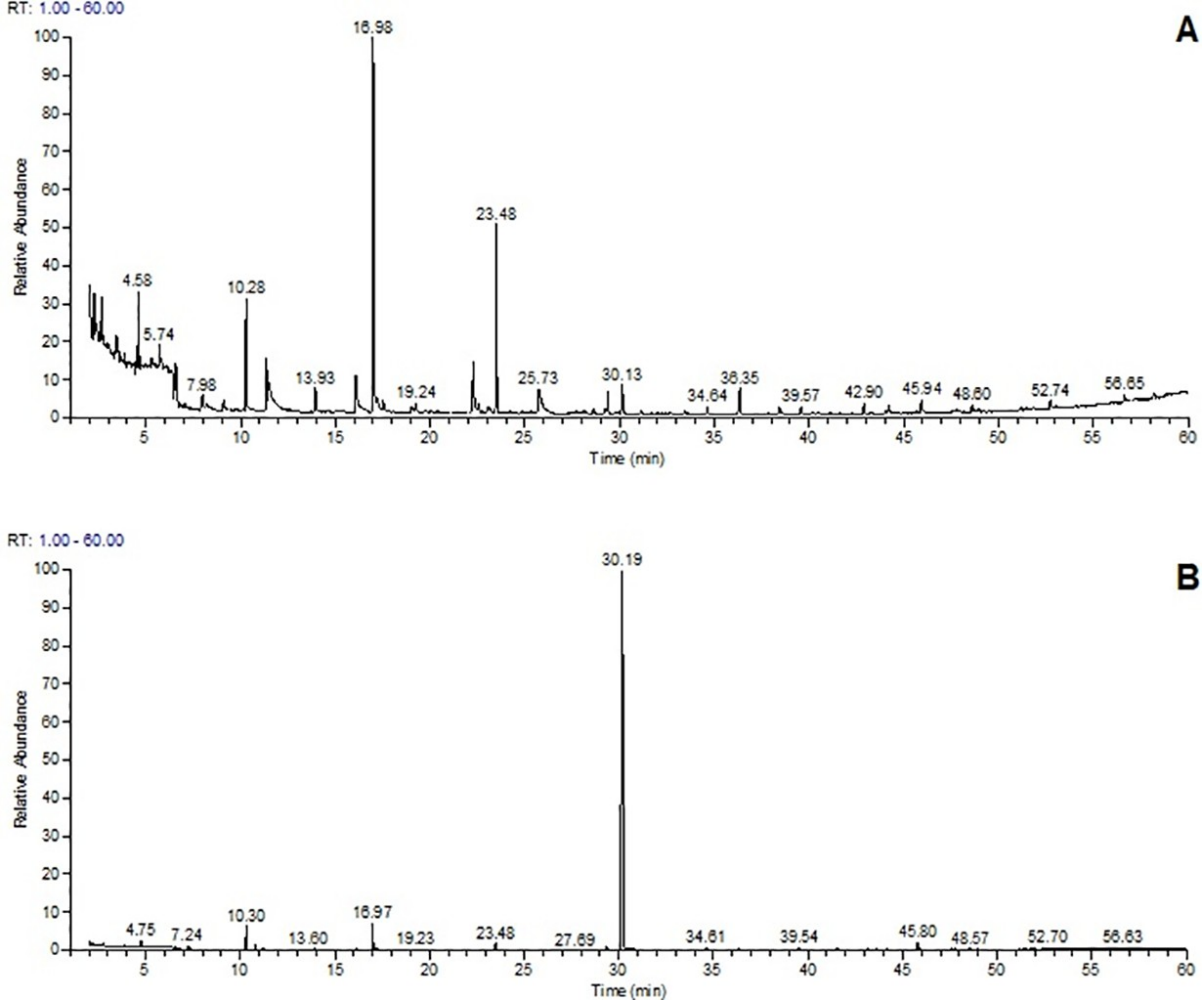
**SPME-GC-MS chromatogram of extracellular media (a), and methanolic extract of *Flavobacterium* sp. biomass (b)**.

Fig 3 shows the comparison of SPME-GC-MS chromatograms of extracellular (a) and intracellular (b) compounds obtained from methanol extract of *Flavobacterium* sp. These results indicate that lupulon can be found in *Flavobacterium* sp. as the intracellular antibiotic constituent, whereas quercetin is secreted into media as an extracellular major compound. In order to confirm the presence of lupulon, one of the major antibiotic constituents in hops, in the extract, we also measured total alpha and beta acids in the same extracts spectrophotometrically. Spectrophotometric measurement of alpha and beta acids in microbial biomass showed 1.06±0% and 0.61±0.1%, respectively (n = 2). These results indicate that intracellular alpha and beta acids can be found in *Flavobacterium* sp. biomass, and lupulon is one of the possible candidate anti-microbial agents as a part of the bacteria’s defense metabolism, however more advanced analytical techniques should be applied to confirm this observation in advance. In order to confirm, the possible extracellular compound found, a confirmation study was performed using other chromatographic techniques.

### 3.3. UPLC results

**Fig 4:**
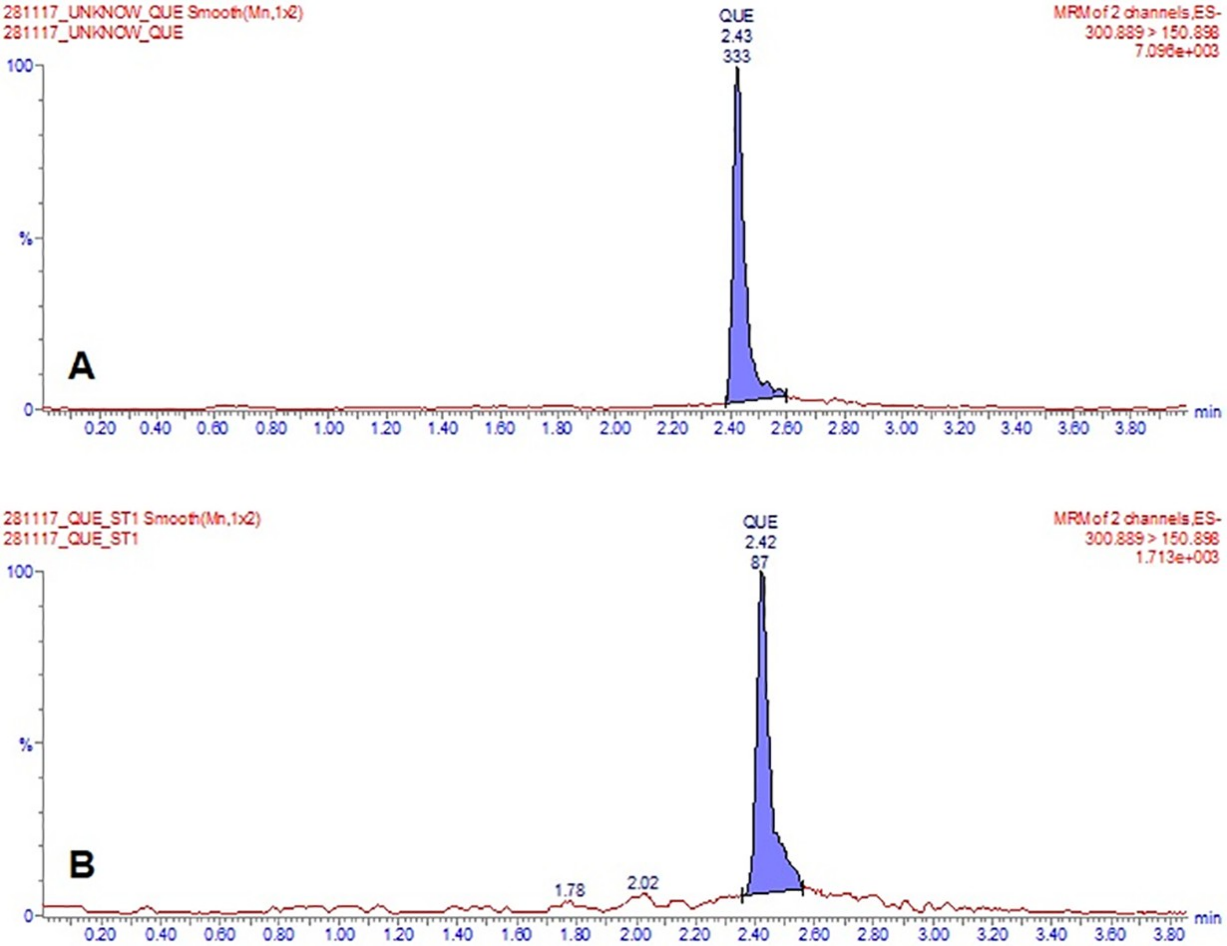
**UPLC chromatogram of methanolic extract of *Flavobacterium* sp. (a) and quercetin as the standard (b)**.

Lyophilized extracellular components were dissolved in absolute methanol for UPLC analysis (20 mg/mL as stock solution). As the standard, quercetin (Sigma-Aldrich, USA) was dissolved in absolute methanol as a final stock concentration of 1 mg/mL. Before running UPLC, an HPLC analysis was conducted to make sure the extract contains quercetin as the major compound, and detected in HPLC. For quercetin, the calibration curves were fitted according to a linear regression (1/X weighing), with correlation coefficients (r^2^) >0.99, indicating a good linearity in quantification process. The retention time of quercetin was 2.43 min respectively. The mean assay values of quercetin in analyte were found to be 0.0002%. Fig 4A and 4B show UPLC chromatogram of methanolic extract of *Flavobacterium* sp. and quercetin as the standard, respectively.

### 3.4. Cytotoxicity results

**Fig 5:**
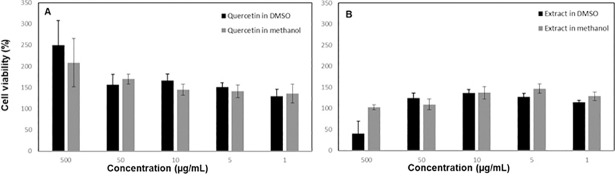
Cytotoxicity of quercetin (A) and *Flavobacterium* sp. (B) extracellular DMSO extract and methanol on ECV-304 cell lines (*P < 0.05).

Cytotoxic activity of *Flavobacterium sp*. extract and quercetin against ECV304 human endothelial cells were shown in Fig 5. Cell viability percentages were increased in both DMSO and methanol extracts of quercetin at the concentration levels of 500 μg/mL (Fig 5A). Interestingly, *Flavobacterium* sp. DMSO extract was more cytotoxic at the concentration level of 500 μg/mL while cytotoxicity results of methanol extract of the *Flavobacterium* sp. at the concentration level of 500 μg/mL were similar when compared to the control (0 μg/mL extract) (Fig 5B). These results indicate that polar solvent, methanol, is more suitable especially for the extraction of Flavobacterium sp. in terms of cell viability in vitro. Methanol is more polar solvent when compared to DMSO.

### 3.5. Computational details results

The quantum chemistry and computation methodologies enable obtaining atomic structures, charges and energetic information of the systems with accuracy equivalent to or greater than those obtained from experiments. Therefore, theoretical calculations have been widely used as an effective tool for intelligent design of new structures and for investigation of the underlying structure-activity relationship. Molecular descriptors characterizing the antioxidant property of any compound, through some parameters such as electronegativity (χ), electron affinity (A), hardness (η), softness (S), electrophilicity index (ω) must be defined by computational methods. These properties are very essential to characterize the antioxidant property of flavonoids [10]. The electronegativity concept (χ) was introduced by Pauling (1960) represents the power of an atom in a molecule to attract an electron towards itself [11]. The chemical hardness (η) is an important quantity in chemical reactivity theory by Pearson et al. (1973), which is the measure of the resistance of a chemical substance towards alteration of its electronic configurations [12]. There is a practical calculation method to calculate for chemical hardness (η) and electronegativity (χ) which was given (Eq 1) by Pearson et al. (1973) [12].

$$\eta \approx \frac{I-A}{2},\;x\approx \frac{I+A}{2}$$ [Eq 1]

I is the ionization potential and A is the electron affinity, there are various techniques to calculate these values. In this study, the Koopman’s theorem was used for the calculation of I and A values derived from the frontier orbital energies of optimized neutral molecules. According to this theorem, the negative of the highest occupied molecular orbital energy (-E_HOMO_) and the lowest unoccupied molecular orbital energy (-E_LUMO_) corresponds to ionization potential and electron affinity, respectively (i.e., I = -E_HOMO_ and A = -E_LUMO_). Using Koopman’s theorem in Eq 2, the chemical hardness and electronegativity are defined in terms of orbital energies:

$$\eta \approx \frac{E(LUMO)-E(HOMO)}{2},\;x=-\mu \approx \frac{-E(LUMO)-E(HOMO)}{2}$$ [Eq 2]

The electrophilicity index (ω) factor being the maximum electron flow between the donor and the acceptor atoms is governed by the decomposition of binding energy between the atoms and it is determined [13]. According to chemical reactivity theory by Pearson et al. (1973) the chemical softness (S) is the measure of the proclivity of a chemical substance towards alteration of its electronic configurations and thus is the inverse of earlier defined chemical hardness coefficient [12]. The ω and S values are calculated by the following Eq 3:

$$\omega \approx \mu 2/2\eta \;S\approx \frac{1}{\left(2\eta \right)}$$ [Eq 3]

**Fig 6:**
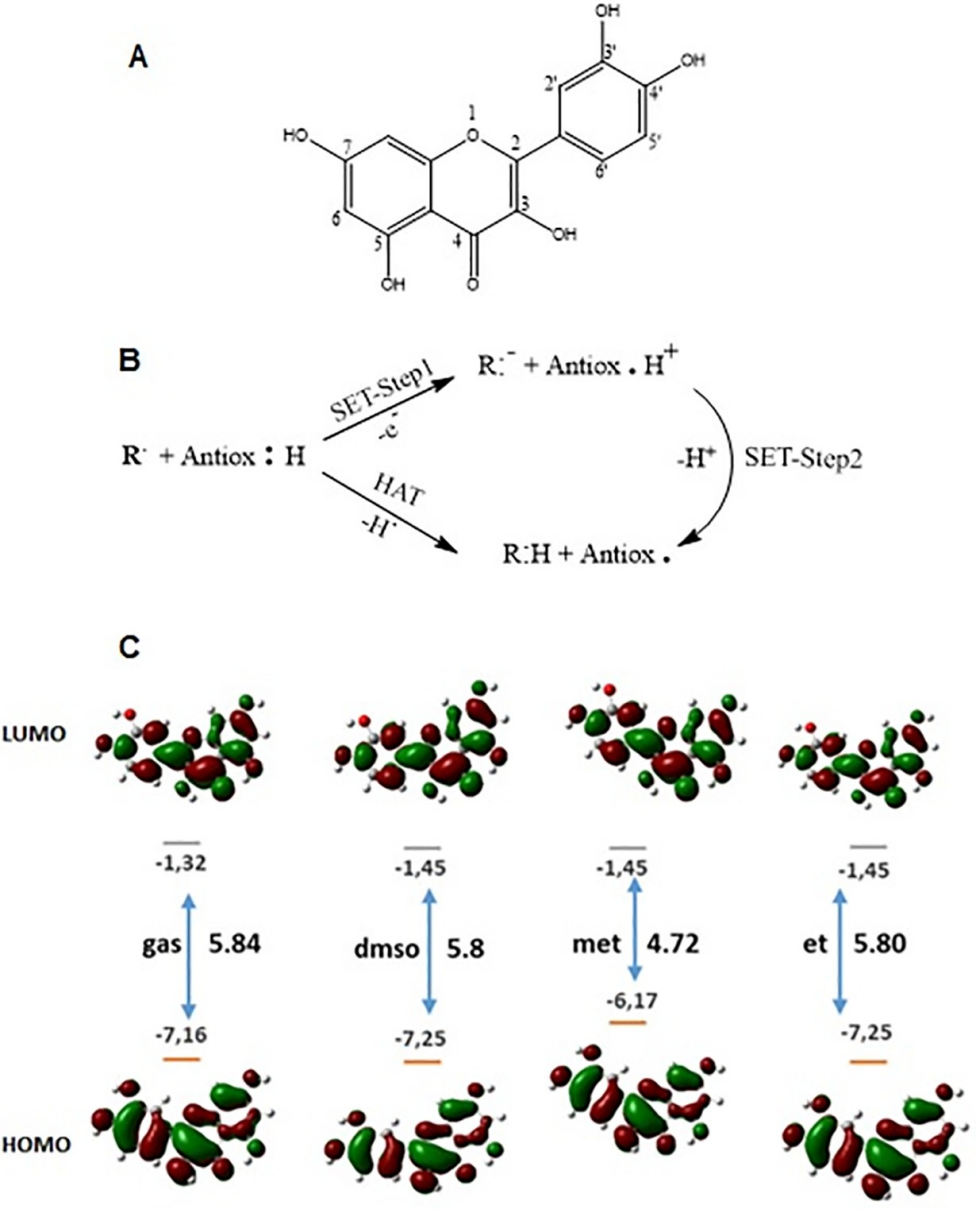
2D structure visulalization and numbering of the atom of the quercetin molecule (A). Two mechanisms of antioxidant, SET and HAT [Liang, N; 2014] (B). HOMO-LUMO energies(eV) and distribution for quercetin in the gas, DMSO, ethanol and methanol solvents (C).

To quantify the antioxidant properties of quercetin molecule (Fig 6A), it is significant to determine χ, A, η, S and ω. These molecular descriptor values obtained from the total energy for the quercetin molecule in gas and as well as in different solvents such as methanol, ethanol and DMSO are listed in Table 3. Calculated χ and ω values show that more polar the solvent is, more it contributes to accentuate the parametric representation of antioxidant property. The calculated ω properties clearly confirm that quercetin prefers to act as electron donor rather than electron acceptor in the studied environments [14,15]. With these results the antioxidant activity levels of quercetin molecule have been quantitatively parameterized. Additionally, we observed that solvent selection has significant effect on electrophile/nucleophile interactions.

**Table 3:** Molecular descriptors values calculated by M062X/6-311++G** level.

Molecular Descriptors				
solvent	χ	η	S	ω
gas	4.238405	2.918079	0.171346	3.078066
DMSO	4.354328	2.900391	0.172391	3.268555
met	3.809435	2.358219	0.212024	3.076855
et	4.349294	2.900799	0.172366	3.260542

*Antioxidant Mechanisms*. Antioxidants are known to scavenge free radicals through a number of mechanisms such as following: (i) hydrogen atom transfer (HAT), (ii) single electron transfer (SET) and (iii) sequential proton loss electron transfer (SPLET). Each mechanism involves different kinetics. The HAT reaction is a concerted movement in which the free radical removes one hydrogen atom of antioxidant, and the antioxidant becomes a radical. In this mechanism, the bond dissociation enthalpies (BDE) that were calculated by Eq 4 (presented as per below) has a numerical value which is the most important parameter in evaluating the antioxidant activity. The weaker O–H bond and lower BDE values are favoring potential antioxidant activity since they enable the reaction of free radical inactivation.

BDE=H(Antiox·)+H(R:H)−H(Antiox:H)−H(R) [Eq 4]

where H(H^.^) is the enthalpy of radical H atom, calculated with the same method that used other calculations in gas phase, methanol, ethanol and DMSO solvents [-0.4981948, -0.4982219, -0.4982216, 0.4982221 au, respectively].

The SET reaction mechanism is initiated by single-electron transfer from the natural flavonoid to the substrate, leading to a radical intermediate (Fig 6B). In SET mechanisms, the antioxidant provides an electron to the free radical and itself then becomes a radical cation (Antiox^.^H^+^). The electron donating ability of the antioxidant is related to an extended electronic delocalization over the entire molecule. The SET–PT mechanism is related with the formation and breaking of flavonoid cation radical which possess a positive charge. In the SET-Step 1 mechanism, the ionization potential (IP) of the antioxidant is the most important energetic factor in evaluating the antioxidant action. The lower the ionization potential, the easier is the electron abstraction.

The calculated ionization potentials (IPs) for quercetin in the gas phase as well as in solvent are given in Table 4. IP values in solvent phases would significantly change with respect to the values in the gas phase as the values are dramatically lower in solvent phases compared to the gas phase. According to the gas phase, the energy drop in the solvent environment varies from about 430 to 490 kj/mol, so this data shows that the polar solvent obtained by increasing the dipole moment (μ_DMSO_ = 3.96>μ_met_ = 1.70>μ_et_ = 1.69 debye) promoted electron donating (IP_DMSO_<IP_met_<IP_et_). The similar results in the literature were determined by Zheng et al. (2017) for the quercetin compound [16]. In this mechanism, the ionization potentials (IPs) were calculated by Eq 5.

IP=H(Antiox.H+)+H(R:−)−H(Antiox:H)−H(R) [Eq 5]

**Table 4:** IP values in kj/mol obtained at DFT/M06-2X/6-311++Gdp level of theory.

Solvent				
IP	gas	DMSO	met	et
	744.7224671	**254.3782727**	308.4238394	314.9378419

SPLET is the third important mechanism for antioxidant activity in which the antioxidant sets the trap for free radicals. In the SPLET reaction mechanism, formation of the Antiox^**-**^ anion is the first step, which is governed by the acid strength of OH group in the compound. The second step of the SPLET reaction mechanism is governed by electron transfer enthalpy (ETE). The net result of SPLET is the same as in HAT mechanism to the free radicals, from an antioxidant point of view.

Antiox:H→Antiox−+H+(associatedwithPA)

Antiox−+R.→Antiox.+R−(associatedwithETE)

R−+H+→RH

The calculated ETE, BDE, PA and PDE values for quercetin in the gas phase as well as in three different solvents (DMSO, met and et) are given in Table 5. Obtained BDE values show that the antioxidant activity the most stable is 4′‒O^.^ radical form in the gas phase and solvent phases. The activity level comparison follows the same sequence as 4′‒O˙>3‒O˙>3′‒O˙>7‒O˙>5‒O˙ in the polar phase. In the literature the same result was obtained by Zheng et al. (2017), but the sequence is not same in gas phase [16]. As a result, it is observed that all solvent studies and the literature data support the oxidation mechanism in polar phase.

**Table 5:** ETE, BDE and PAvalues in kj/mol obtained at DFT/M06-2X/6-311++Gdp level of theory.

		Bond				
	solvent	3′‒OH	4′‒OH	3‒OH	5‒OH	7‒OH
**BDE**	**gas**	437.4486	**404.3757**	428.1282	**408.2813**	482.045
	**DMSO**	425.1334	409.0267	412.8339	455.9413	448.4485
	**met**	**424.5807**	408.1406	**412.3256**	455.5462	**447.6296**
	**et**	426.3975	409.6214	414.1878	457.5226	449.1776
**ETE**	**gas**	**187.3874**	230.2574	204.5535	264.9334	288.3545
	**DMSO**	71.03632	84.76479	**70.4742**	109.9867	121.8466
	**met**	71.88619	86.0652	**71.5058**	111.107	122.991
	**et**	72.77335	87.4092	**72.5809**	112.2672	124.1749
**PA**	**gas**	194.3064	**118.3641**	167.8202	180.8198	137.9372
	**DMSO**	-32.1049	**-61.9398**	-43.84296	-40.2473	-59.5993
	**met**	-30.1416	**-60.7605**	-42.0169	-38.3969	-58.1967
	**et**	-25.7523	**-57.1641**	-37.7702	-34.1201	-54.3730

According to the mechanism of SPLET, the formation of flavonoid radical is the second step, which is governed by electron transfer enthalpy (ETE). Table 5 presents the calculated ETE values in gas phase as well as in other solvents for quercetin. While, the lowest ETE was found for 3′–O· radical formation in the gas phase, for all other solvent phases 3–O· radical formation was found to have the lowest ETE. This result we obtained is consistent with the results by Zheng et al. (2017) in literature [16]. Zheng et al. (2017) found that the ETE value of the radical formation in position 3′–O· was more stable than the position 3–O· when they went into the ethanol and water phases [16]. According to the obtained results, 7–O· radical formation was found the biggest ETE value in the all studied phases including gas phase, this inference is consistent with the literature. These results did not show which ring had more effect, but A ring showed little contribution. Another important result was obtained that the lowest ETE values of the radical formation for each group in the DMSO solvent, followed by ethanol and methanol solvents, respectively.

According to the SPLET mechanism, proton affinities (PAs) were calculated in the gas phase and as well as in solvents to investigate the deprotonation of the phenolic OH group(s). Table 5 displays that the PA values of 4′‒OH are always the smallest in all studied environments and this demonstrates that the formation of 4′-O^-^ anion occurs most easily as compared with other anion positions. The other result from the PA values table is that polar solvents significantly decrease PA values when compared to the gas solvents. We can also observe that proton affinity values for different type of solvents follow the same gas>et>met>DMSO magnitude comparison sequence concerning the quercetin molecule behavior in each of these solvents. Whereas the dipole moment values exhibit reverse order gas<et<met<DMSO magnitude comparison sequence being consistent with the fact that the increase of polarity decreasing the proton affinity. The PA results obtained from our study are consistent with the study by Zheng et al. (2017) for the quercetin molecule, and the polar solvent propensity of PA was also found in the work by Liang et al (2015) [16,17].

Antioxidant activity has been found to be related to the HOMO-LUMO energy values of frontier orbitals, and their distribution. The obtained frontier energy values and their distribution for quercetin molecule in the gas, DMSO, ethanol and methanol solvents are depicted in Fig 6C.

It can be seen from the Fig 6C that similar distribution of HOMO-LUMO orbitals are determined in all studied environments. An important point that needs to be emphasized is that in the methanol phase the HOMO energy is the highest (-6.17 eV). Therefore HOMO-LUMO band gap (4.72 eV) is smaller in methanol when compared to the other environment which shows that the quercetin molecule in methanol has stronger electron donating ability.

## Discussion

Production of flavonoids by microorganisms was reported, previously. Genetically-engineered microorganisms such as *Escherichia coli* BL21 can be used for the production of flavonoids such as astilbin, quercitrin for industry [18,19]. Conversion of rutin to quercetin using microorganisms such as *Paenibacillus glucanolyticus* is one of the approaches to produce quercetin [20]. Fermentation of litchi pericarp by *Aspergillus awamori* led to the production of quercetin [21]. *Pyrococcus furiosus* β-glucosidase transforms rutin to quercetin [22]. Quercetin, a polyphenolic flavonoid, production by bacteria such as *Paenibacillus glucanolyticu* D3 was previously reported [20], however but the number of articles about quercetin-producing bacteria is very limited.

Various strains of *Flavobacterium* sp. were identified as gram-negative, aerobic forming yellow colonies such as *Flavobacterium seoulense*, *Flavobacterium verecundum* [22,23]. Oil degrading strains such as *Flavobacterium naphthae* sp. nov. forms also yellow colonies [24]. Most of *Flavobacterium* genus members are safe, while some are opportunistic pathogens which may lead diseases in aquatic organisms [25]. *Flavobacterium cheonhonense* sp. nov. designated strain ARSA-15(T) produces deep-yellow pigment, and is catalase- and oxidase-positive [26]. The isolated bacterium in our study which is the highest similarity to *Flavobacterium cheonhonense* ARSA-15(T) also produces yellow colored morphology.

The *Flavobacterium* genus includes very interesting representative species producing industrially important compounds. For example, different fungistatic and bacteriostatic agents such as flavocin can be produced by members of this genus such as *Flavobacterium* sp. L-30 [27]. Several strains such as *Flavobacterium* sp. YS-80-122 can secrete industrially important enzymes such as ι-carrageenans and heparinase II (Hep II) is produced by *Flavobacterium heparinum* [28,29]. Some *Flavobacterium* strains such as *Flavobacterium* sp. 200Cs-4 has tolerance against toxic heavy metals such as cesium [30]. Quercetin-producer microorganisms in literature are very limited, and it is not known that any *Flavobacterium* strain isolated can produce quercetin. However, in this study we demonstrated that the *Flavobacterium* isolate produces quercetin efficiently for the first time.

Quercetin has dual role showing both cytoprotective and cytotoxic effects in biological systems [31]. It has protective effect against oxidative stress in vitro and in vivo blood cells [32]. Apoptosis, programmed cell death, in cardiomyocytes is reduced by quercetin via the suppression of tumor necrosis factor-alpha increase in posttraumatic cardiac dysfunction [33]. Quercetin also protects against acute liver injury, hepatic fibrosis, and lung carcinogenesis [34–36]. Quercetin reverses preneoplastic lesions by epidermal growth factor receptor modulation [37]. It can be used as a protective agent against coronary diseases [38]. It has been shown that quercetin is a promising therapeutic agent for improving retinal ganglion cells (RGC) survival and function in glaucomatous neurodegeneration [39]. Low concentration of quercetin antagonizes glioblastoma cell invasion and angiogenesis in vitro [40].

The possible mechanism for quercetin-induced apoptosis in cancer cell line is the direct interaction with DNA by triggering the intrinsic pathway [41]. Apoptotic induction can be also induced by modulation of p53 posttranslational modifications (Zhang and Zhang, 2017), and modulation of NF-κB signaling [42,43]. Quercetin has a selective toxic effect on HuH7 and HepG2 liver cancer cells and normal human epithelial cell lines. Hepatocellular carcinoma cells undergo apoptosis through intrinsic pathway due to arrested cells in S phase by quercetin [37]. Cytotoxicity of quercetin against leukemic cells and breast cancer cells, but not healthy cells was reported [41]. Although quercetin production by *Flavobacterium* sp. has not been reported, yet, direct cytotoxic activity of *Flavobacterium* sp. against apoptosis has been shown. For example, *Flavobacterium psychrophilum* induces rainbow trout muscle apoptosis through the modulation of the NF-κB signaling [44]. As observed in our study, DMSO extract of the *Flavobacterium* isolate showed cytotoxicity against ECV304 cell line. The type of solvent such as methanol and DMSO also effects the cytotoxic profile of not only quercetin but also *Flavobacterium* sp. extract indicating that methanol, a polar solvent, results in a higher cell viability results. The cytotoxicity results are confirmed by computational methods indicating that quercetin and *Flavobacterium* sp. extracts show better cell viability when extracted in methanol when compared to DMSO. The quercetin produced by *Flavobacterium* sp. can be used for industrial purposes with a suitable extraction process in advance in future.

## Conclusions

This study reports for the first time production of quercetin by a *Flavobacterium sp*. identified as *Flavobacterium cheonhonense* strain ARSA-15 (99%). Quercetin is detected as the major extracellular compound produced by the *Flavobacterium* sp. Type of solvent affects the cytotoxicity of the quercetin showing that the methanol extract of *Flavobacterium* sp. resulted in a higher cell viability results when compared to its DMSO extract which was confirmed by computational chemistry. Since the quercetin molecule found in *Flavobacterium* sp. extract in methanol has stronger electron donating ability based on the computational results, it was shown that *Flavobacterium* sp. methanol extract led to a better cell viability results possibly because of enhanced antioxidant capacity of the quercetin molecule available in the extract. In conclusion, *Flavobacterium* sp. can be used as a bioresource to produce quercetin for industrial purposes.

## Supporting information

S1 Fig Graphical abstract. (TIF)

S1 Supporting Information Data set is available here. (ZIP)

## References

[1] Brown DJ, Britton G & Goodwin TW (1975) Carotenoid biosynthesis by a cell-free preparation from a *Flavobacterium* species. *Biochem Soc Trans* 3(5):741–2. 1193283 10.1042/bst0030741

[2] Takahashi K, Abe JI, Kozuma T, Yoshida M, Nakamura N. & Hizukuri S. (1996). Production and application of an isoamylase from *Flavobacterium odoratum*, *Enzyme and Microbial Technology* 19, 456–461.

[3] Nam HK, Hong SH, Shin KC, & Oh DK. (2012). Quercetin production from rutin by a thermostable β-rutinosidase from *Pyrococcus furiosus*. *Biotechnol*. *Lett*. 34, 483–489. 10.1007/s10529-011-0786-2 22052256

[4] Singh PD, Young MG, Johnson JH, Cimarusti CM, & Sykes RB. (1984). Bacterial production of 7-formamidocephalosporins. Isolation and structure determination. *J*. *Antibiot*. *(Tokyo)* 37(7):773–80. 6547947 10.7164/antibiotics.37.773

[5] Shoji J, Sakazaki R, Kato T, Terui Y, Matsumoto K, Tanimoto T. et al (1985). Isolation of chitinovorin D. *J*. *Antibiot*. *(Tokyo)* 38(4):538–40. 3839232 10.7164/antibiotics.38.538

[6] ASBC Methods of Analysis (http://mountainhort.ncsu.edu/fletcher/programs/nchops/images/UV_VIS_method.pdf)

[7] Pirildar S, Sütlüpınar N, Atasever B, Erdem-Kuruca S, Papouskova B. & Šimánek V., (2010). Chemical constituents of the different parts of *Colchicum baytopiorum* (Liliaceae) and their cytotoxic activities on K562 and HL60 cell-lines. *Pharm*. *Biol*. 48, 32–39. 10.3109/13880200903029373 20645753

[8] Dennington R, Keith T, Millam J. (2009). GaussView, Version 5 Semichem Inc. Shawnee Mission.

[9] Frisch A, Dennington IIR, Keith T, Millam J, Nielsen AB, Holder AJ. et. al. Reference, Version 4.0, Gaussian Inc., Pittsburgh, 2007., Frisch MJ (2009) Gaussian 09 revision A.1. Gaussian Inc., Wallingford, CT.

[10] Parr RG. & Pearson RG. (1983). Absolute hardness: companion parameter to absolute electronegativity. *J*. *Am*. *Chem*. *Soc*. 105, 7512–7516.

[11] Pauling L. (1960). The Nature of the Chemical bond, 3rd ed; Cornell University Press: Ithaca, NY,.

[12] Pearson RG. (1973). Hard and Soft Acids and Bases; Dowen, Hutchinson and Ross: Stroudsberg,.

[13] Parr RG, Szentpaly LV. & Liu S. (1999). Electrophilicity index. *J*. *Am*. *Chem*. *Soc*. 121, 1922–1924.

[14] Jeevitha D, Sadasivam K, Praveena R. & Jayaprakasam R. (2016). DFT study of glycosyl group reactivity in quercetin derivatives. *J*. *Mol*.*Struct*. 1120, 15–24.

[15] Praveena R, Sadasivam K, Deepha V. & Sivakumar R. (2014). Antioxidant potential of orientin: a combined experimental and DFT approach. *J*. *Mol*. *Struct*. 1061, 114–123.

[16] Zheng YZ, e G, Liang Q, Chen DF, Guo R. & Lai RC. (2017). Antioxidant activity of quercetin and its glucosides from propolis: a theoretical study. *Sci*. *Rep*. 7: 7543 10.1038/s41598-017-08024-8 28790397 PMC5548903

[17] Liang N, Kitts DD. (2014). Antioxidant Property of Coffee Components: Assessment of Methods that Define Mechanisms of Action, *Molecules* 19, 19180–19208. 10.3390/molecules191119180 25415479 PMC6270823

[18] Thuan NH, Malla S, Trung NT, Dhakal D, Pokhrel AR, Chu LL. et al (2017). Microbial production of astilbin, a bioactive rhamnosylated flavanonol, from taxifolin. *World J*. *Microbiol*. *Biotechnol*. 33, 36 10.1007/s11274-017-2208-7 28120309

[19] De Bruyn F, Van Brempt M, Maertens J, Van Bellegem W, Duchi D. & De Mey M., (2015). Metabolic engineering of *Escherichia coli* into a versatile glycosylation platform:production of bio-active quercetin glycosides. *Microb*. *Cell Fact*. 14, 138 10.1186/s12934-015-0326-1 26377568 PMC4573293

[20] Lu ZT, Zhang YT. & Nan Shi N. (2012). Screening and identification of a quercetin-producing bacterium Paenibacillus glucanolyticu D3. *Environment and Natural Resources Research* 2, 106–113.

[21] Lin S, Zhu Q, Wen L, Yang B, Jiang G, Gao H. et al (2014). Production of quercetin, kaempferol and their glycosidic derivatives from the aqueous-organic extracted residue of litchi pericarp with *Aspergillus awamori*. *Food Chem*. 145, 220–227. 10.1016/j.foodchem.2013.08.048 24128471

[22] Ekwe AP. & Kim SB. (2017). *Flavobacterium commune* sp. nov., isolated from freshwater and emended description of *Flavobacterium seoulense*. *Int*. *J*. *Syst*. *Evol*. *Microbiol*. 10.1099/ijsem.0.002463 29111965

[23] Chen WM, Su C.L. & Sheu SY. (2017). *Flavobacterium lacunae* sp. nov., isolated from a freshwater pond. *Int*. *J*. *Syst*. *Evol*. *Microbiol*. 67, 875–882. 10.1099/ijsem.0.001693 29162201

[24] Chaudhary DK. & Kim J. (2017). *Flavobacterium naphthae* sp. nov., isolated from oil-contaminated soil. *Int*. *J*. *Syst*. *Evol*. *Microbiol*. 10.1099/ijsem.0.002504 29185939

[25] Waśkiewicz A. & Irzykowska L. (2014). *Flavobacterium* spp.–characteristics, occurrence, and toxicity. Encyclopedia of Food Microbiology (Second Edition), pages 938–942.

[26] Lee S, Oh JH, Weon HY. & Ahn TY. (2012). *Flavobacterium cheonhonense* sp. nov., isolated from a freshwater reservoir. *J*. *Microbiol*. 50, 562–566. 10.1007/s12275-012-1229-z 22923102

[27] Shenin IuD, Kruglikova LF, Vasiuk LF, Kozhemiakov AP, Chebotar VK & Popova TA. (1996). A new metabolite with fungistatic and bacteriostatic activity, produced by strain L-30 of *Flavobacterium* sp. *Antibiot*. *Khimioter*. 41, 6–12. 9054320

[28] Li S, Hao J. & Sun M, (2017). Cloning and characterization of a new cold-adapted and thermo-tolerant ι-carrageenase from marine bacterium *Flavobacterium* sp. YS-80-122. *Int*. *J*. *Biol*. *Macromol*. 102, 1059–1065. 10.1016/j.ijbiomac.2017.04.070 28435055

[29] Zhou B, Cheng Y, Deng C, Liu W, Chen C, Chen J. et al (2014). Culture conditions optimization and high cell density fermentation of recombinant bacteria producing heparinase II from *Flavobacterium heparinum*. *Sheng Wu Gong Cheng Xue Bao*. 30, 674–678. 25195257

[30] Kato S, Goya E, Tanaka M, Kitagawa W, Kikuchi Y, Asano K. et al (2016). Enrichment and isolation of *Flavobacterium* strains with tolerance to high concentrations of cesium ion. *Sci*. *Rep*. 6: 20041 10.1038/srep20041 26883718 PMC4756683

[31] Nishimura K, Matsumoto R, Yonezawa Y. & Nakagawa H. (2017). Effect of quercetin on cell protection via erythropoietin and cell injury of HepG2 cells. *Arch*. *Biochem*. *Biophys*. 636, 11–16. 10.1016/j.abb.2017.10.013 29080630

[32] Bustos PS, Deza-Ponzio R, Páez PL, Albesa I, Cabrera JL, Virgolini MB et al (2016). Protective effect of quercetin in gentamicin-induced oxidative stress in vitro and in vivo in blood cells. Effect on gentamicin antimicrobial activity. *Environ Toxicol Pharmacol* 48, 253–264. 10.1016/j.etap.2016.11.004 27846408

[33] Jing Z, Wang Z, Li X, Li X, Cao T, Bi Y, et al (2016). Protective effect of quercetin on posttraumatic cardiac injury. *Sci*. *Rep*. 6: 30812 10.1038/srep30812 27470932 PMC4965739

[34] Wu L, Zhang Q, Mo W, Feng J, Li S, Li J. et al (2017). Quercetin prevents hepatic fibrosis by inhibiting hepatic stellate cell activation and reducing autophagy via the TGF-β1/Smads and PI3K/Akt pathways. *Sci*. *Rep*. 7: 9289 10.1038/s41598-017-09673-5 28839277 PMC5571156

[35] Wang X, Wang L, Zhang H, Li K. & You J. (2016). Ultrastructural changes during lung carcinogenesis-modulation by curcumin and quercetin. *Oncol*. *Lett*. 12, 4357–4360. 10.3892/ol.2016.5259 28101199 PMC5228324

[36] Peng Z, Gong X, Yang Y, Huang L, Zhang Q, Zhang P. et al (2017). Hepatoprotective effect of quercetin against LPS/d-GalN induced acute liver injury in mice by inhibiting the IKK/NF-κB and MAPK signal pathways. *Int*. *Immunopharmacol*. 52, 281–289. 10.1016/j.intimp.2017.09.022 28963941

[37] Carrasco-Torres G, Monroy-Ramírez HC, Martínez-Guerra AA, Baltiérrez-Hoyos R, Romero-Tlalolini MLÁ, Villa-Treviño S. et al (2017) Quercetin reverses rat liver preneoplastic lesions induced by chemical carcinogenesis. *Oxid Med Cell Longev*. 10.1155/2017/4674918 28740570 PMC5504959

[38] Griffiths K, Aggarwal BB, Singh RB, Buttar HS, Wilson D. & De Meester F. (2016). Food antioxidants and their anti-inflammatory properties: a potential role in cardiovascular diseases and cancer prevention. *Diseases* 4, pii: E28. 10.3390/diseases4030028 PMC5456284 28933408

[39] Gao FJ, Zhang SH, Xu P, Yang BQ, Zhang R, Cheng Y et al (2017). Quercetin declines apoptosis, ameliorates mitochondrial function and improves retinal ganglion cell survival and function in vivo model of glaucoma in rat and retinal ganglion cell culture in vitro. *Front*. *Mol*. *Neurosci*. 10, 285 10.3389/fnmol.2017.00285 28936163 PMC5594060

[40] Liu Y, Tang ZG, Yang JQ, Zhou Y, Meng LH, Wang H. et al (2017). Low concentration of quercetin antagonizes the invasion and angiogenesis of human glioblastoma U251 cells. *Onco*. *Targets Ther*. 10, 4023–4028. 10.2147/OTT.S136821 28860810 PMC5565384

[41] Srivastava S, Somasagara RR, Hegde M, Nishana M, Tadi SK, Srivastava M. et al (2016). Quercetin, a natural flavonoid interacts with DNA, arrests cell cycle and causes tumor regression by activating mitochondrial pathway of apoptosis. *Sci*. *Rep*. 6:24049 10.1038/srep24049 27068577 PMC4828642

[42] Zhang P. & Zhang X. (2017). Stimulatory effects of curcumin and quercetin on posttranslational modifications of p53 during lung carcinogenesis. *Hum*. *Exp*. *Toxicol*. 10.1177/0960327117714037 28681665

[43] Zhang W, Yin G, Dai J, Sun YU, Hoffman RM, Yang Z. et al (2017). Chemoprevention by quercetin of oral squamous cell carcinoma by suppression of the NF-κB signaling pathway in DMBA-treated hamsters. *Anticancer Res*. 37, 4041–4049. doi: 10.21873/anticanres.11789 28739686 10.21873/anticanres.11789

[44] Iturriaga M, Espinoza MB, Poblete-Morales M, Feijoo CG, Reyes AE, Molina A, et al (2017). Cytotoxic activity of *Flavobacterium psychrophilum* in skeletal muscle cells of rainbow trout (Oncorhynchus mykiss). *Veterinary Microbiology* 210, 101–106. 10.1016/j.vetmic.2017.09.009 29103678

