# Supplementary figures and images for: Chemical and molecular characterization of metabolites from Flavobacterium sp
Source: PLoS One. 2018 Oct 17;13(10):e0205817. doi: 10.1371/journal.pone.0205817 (PMC6192653; doi:10.1371/journal.pone.0205817)

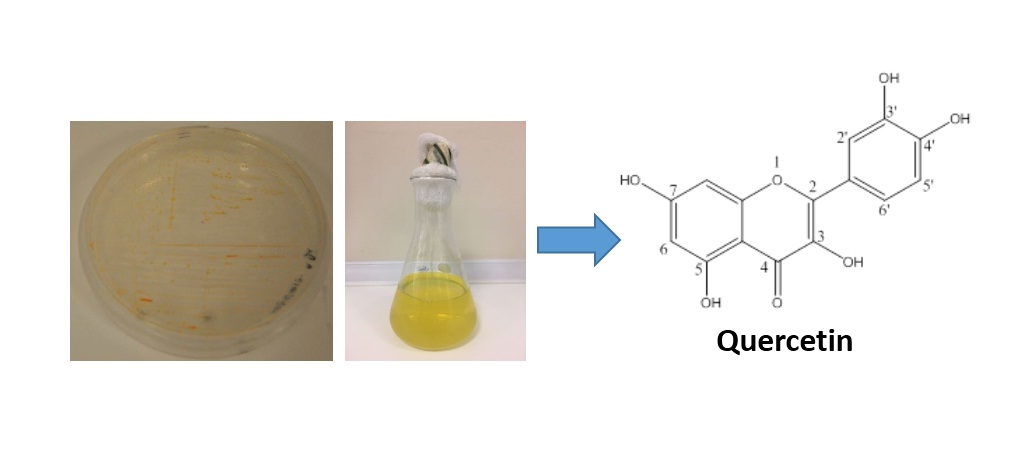

Supplement: S1 Fig — (TIF) [file pone.0205817.s001.tif]
