# Supplementary material for: Chemical and molecular characterization of metabolites from Flavobacterium sp
Source: PLoS One. 2018 Oct 17;13(10):e0205817. doi: 10.1371/journal.pone.0205817 (PMC6192653; doi:10.1371/journal.pone.0205817)
Supplement: S1 Supporting Information — (ZIP) [file pone.0205817.s002.zip › Data Set/gc-ms/EHDOSYA.pdf]

TÜBİTAK
MAM
TÜRKİYE BİLİMSEL VE TEKNİK ARAŞTIRMA KURUMU
MARMARA ARAŞTIRMA MERKEZİ
GIDA ENSTİTÜSÜ

P.K 21, 41470 GEBZE/KOCAELİ
T 0 262 677 20 00 F 0 262 641 23 09
http://mam.tubitak.gov.tr

TEST/ANALİZ/ÖLÇÜM RAPORU

(Endüstriyel Teknik Destek Hizmeti)

**Rapor No**	:	49362558-125.05- 977 / **2745**
**Rapor Tarihi**	:	11/05/2017
**Talep Eden**	:	ÜSKÜDAR ÜNİVERSİTESİ.
**Adres**	:	HALUK TÜRKSOY SOK. NO:14 34662 ALTUNİZADE İSTANBUL
**Konusu**	:	KİMYASAL ANALİZLER

Bu raporda incelenen sonuçlar sadece incelenen numunelere aittir.

Onaylayan

Neşe Aslı ÖNCÜ
Gıda Enstitüsü Endüstriyel Hizmet Sorumlusu

Bu rapor ve sonuçları talepte bulunan kuruluş ve müşterilerince ticaret ve reklam amaçları ile kullanılamaz. Rapor tamamen veya kısmen çoğaltılamaz/yayınlanamaz.
Raporda (*) işaretli analizler akredite edilmiştir.
İmzasız analiz raporları geçersizdir.

SGS SGS

**Rapor No**	: 49362558-125.05-	977	,2745
**Talep Eden**	: ÜSKÜDAR ÜNİVERSİTESİ.		
**Adres**	: HALUK TÜRKSOY SOK. NO:14 34662 ALTUNİZADE İSTANBUL		

Örnek: EKSTRAKIT(METANOL %0,1 BHT İÇEREN)

**Parti / Lot No**	:	**Son kullanım Trh**	:
**Örnek Sayısı**	: 1	**Üretim Tarihi**	:
**Örneğin getiriliş şekli**	: Elden	**Enstitü örnek kayıt no**	: 17-973/001
**Kabul anındaki durumu**	: Plastik ambalaj	**Kabul tarihi ve saati**	: 26/04/2017 16:00:00
		**Analiz Tarihi**	: 10/05/2017 - 10/05/2017

Şahit numune bilgileri : ( ) Müşteriye geri iade ( ) Şahit numune mevcut (x) Şahit numune alınmamıştır

Analiz	Sonuç	Yöntem
SPME-GC-MS yöntemi ile kalitatif bileşen analizi	Sonuçlar ektedir.	SPME-GC-MS

Açıklamalar:

Sorumlu İmzalar:

53629

Bu rapor ve sonuçları talepte bulunan kuruluş ve müşterilerince ticaret ve reklam amaçları ile kullanılamaz. Rapor tamamen veya kısmen çoğaltılamaz/yayınlanamaz. Raporda (*) işaretli analizler akredite edilmiştir. İmzasız analiz raporları geçersizdir.

Bu rapor 3 sayfa olup , 2 asıl (1 asıl müşteriye, 1 asıl Enstitü arşivine) olarak hazırlanmıştır.

P.K 21, 41470 GEBZE/KOCAELİ
T 0 262 677 20 00 F 0 262 641 23 09
http://mam.tubitak.gov.tr

TÜBİTAK
MAM

Rapor No : 49362558-125.05- 977 ,2745
Talep Eden : ÜSKÜDAR ÜNİVERSİTESİ.
Adres : HALUK TÜRKSOY SOK. NO:14 34662 ALTUNİZADE İSTANBUL

Örnek: SOLVENT:SU		Son kullanım Trh	:
		Üretim Tarihi	:
Parti / Lot No	:	Enstitü örnek kayıt no	: 17-973/002
Örnek Sayısı	: 1	Kabul tarihi ve saati	: 26/04/2017 16:00:00
Örneğin getiriliş şekli	: Elden	Analiz Tarihi	: 10/05/2017 - 10/05/2017
Kabul anındaki durumu	: Plastik ambalaj		

Şahit numune bilgileri : ( ) Müşteriye geri iade ( ) Şahit numune mevcut (x) Şahit numune alınmamıştır

Analiz	Sonuç	Yöntem
SPME-GC-MS yöntemi ile kalitatif bileşen analizi	Sonuçlar Ektedir.	SPME-GC-MS

Açıklamalar:

Sorumlu İmzalar:

53629

Bu rapor ve sonuçları talepte bulunan kuruluş ve müşterilerince ticaret ve reklam amaçları ile kullanılamaz. Rapor tamamen veya kısmen çoğaltılamaz/yayınlanamaz. Raporda (*) işaretli analizler akredite edilmiştir. İmzasız analiz raporları geçersizdir.

Bu rapor 3 sayfa olup , 2 asıl (1 asıl müşteriye, 1 asıl Enstitü arşivine) olarak hazırlanmıştır.

P.K 21, 41470 GEBZE/KOCAELİ
T 0 262 677 20 00 F 0 262 641 23 09
http://mam.tubitak.gov.tr
