# Supplementary material for: Chemical and molecular characterization of metabolites from Flavobacterium sp
Source: PLoS One. 2018 Oct 17;13(10):e0205817. doi: 10.1371/journal.pone.0205817 (PMC6192653; doi:10.1371/journal.pone.0205817)
Supplement: S1 Supporting Information — (ZIP) [file pone.0205817.s002.zip › Data Set/UPLC/Quercetin_aYKUT 27112017.docx]

*Instrument Parameters*

Chromatographic separation was achieved using a Waters Acquity UPLC System consisting of a sample manager, a column heater/cooler, a binary solvent manager, and 10.0 μL injection loop. The chromatographic separation was achieved using an ACQUITY UPLC® HSS C18 (1.8 μm, 2.1 x 150 mm) column. The acquity auto-sampler temperature was set at 10°C and the injection volume was 5.0 μL. The column was maintained at 50°C. Mobile phase A for both the loading and eluting pumps consisted of 5.0 mM ammonium formate pump (pH=3.0) while mobile phase B consisted of acetonitrile with 0.1% formic acid. The flow rate of the mobile phase under gradient condition was kept at 0.5 mL/min. The gradient consisted of linear gradient from 4% B to 50%B (0–2 min), back to 4 % B (2–3 min) and held constant at 95 %B (3–4 min). The total run time was 4.0 min.

The detection of Quercetin was performed using a Waters Xevo TQD (triple quadrupole) tandem mass spectrometer (Waters Corp., Milford, MA, USA) with an electro-spray ionization source in negative ionization mode. Quantitation was performed using multiple reaction monitoring (MRM) mode to study parent → product ion (m/z) transitions Quercetin (301.0 /150.9). The MS/MS instrument parameters were optimized, including capillary voltage 3.0 kV, nitrogen gas temperature 450°C, source temperature 150°C, collision gas flow 0.22 mL/min, nitrogen gas flow 20 L/h, and de-solvation gas flow 1,000 L/h.

*Preparation of Reagents and Standards*

Calibration standards for Quercetin: 6.25, 12.5, 25.0, 50.0 100.0, and 250.0 ng/mL and were prepared by spiking methanol.

TABLES

Unknow samples

The lower limits of quantification (LLOQ) level concentration sample for Quercetin
