# Supplementary material for: Chemical and molecular characterization of metabolites from Flavobacterium sp
Source: PLoS One. 2018 Oct 17;13(10):e0205817. doi: 10.1371/journal.pone.0205817 (PMC6192653; doi:10.1371/journal.pone.0205817)
Supplement: S1 Supporting Information — (ZIP) [file pone.0205817.s002.zip › Data Set/gc-ms/EHDOSYA 1.doc]

**QUAN PEAK RESULT**

Data File:

1740000407 02

Sample Name:

Solvent: Su

Acquisition Date:

05/09/17 02:36:42 PM

Instrument Method:

C:\Xcalibur\EH2017\17400040701 02\Aroma.meth

Current Processing Method:

C:\Xcalibur\EH2017\17400040701 02\process

Current Data Path:

C:\Xcalibur\EH2017\17400040701 02

RT

Peak Area

Area %

2.27

5804994

2.39

2.66

7727503

3.18

3.46

6093673

2.51

4.60

8281065

3.40

5.31

4438154

1.82

5.75

15591723

6.41

6.34

23820164

9.79

6.56

6049787

2.49

8.00

5202953

2.14

9.11

2110027

0.87

10.28

11896447

4.89

11.37

16133759

6.63

13.95

3577832

1.47

16.10

12184559

5.01

16.99

47679744

19.60

17.53

1700390

0.70

19.24

3227526

1.33

22.27

12397500

5.10

23.49

23900217

9.83

25.75

9794060

4.03

29.38

3877534

1.59

30.13

5509526

2.26

36.34

3972636

1.63

45.94

2277911

0.94

RT

Compound Name

SI

RSI

2.27

Cyclotetrasiloxane, octamethyl- (CAS)

752

779

2.27

Cyclotetrasiloxane, octamethyl-

751

788

2.27

Cyclotetrasiloxane, octamethyl- (CAS)

750

798

2.27

Cyclotetrasiloxane, octamethyl- (CAS)

725

731

2.27

Cyclotetrasiloxane, octamethyl- (CAS)

722

791

Hit Spectrum

RT

Compound Name

SI

RSI

2.66

Silicic acid, diethyl bis(trimethylsilyl) ester

722

776

2.66

(S)-(E)-(-)-4-Acetoxy-1-phenyl-2-dodecen-1-one

722

898

2.66

Cyclotrisiloxane, hexamethyl- (CAS)

704

855

2.66

Cyclotrisiloxane, hexamethyl-

698

898

2.66

Cyclotrisiloxane, hexamethyl- (CAS)

690

807

Hit Spectrum

RT

Compound Name

SI

RSI

3.46

6,7-Dimethoxy-2-methyl-3,4-dihydro[1-D]isoquinolinium ion

745

864

3.46

1,2-Dihydro-1,4-diphenylphthalazine

715

768

3.46

4-(2',4'-dimethoxy-6'-propylbenzoyloxy)-2-hydroxy-6-pentylbenzoic acid

705

931

3.46

2-(5-CHLORO-2-METHOXYPHENYL)PYRROLE

703

802

3.46

Cyclotrisiloxane, hexamethyl- (CAS)

691

826

Hit Spectrum

RT

Compound Name

SI

RSI

4.60

1,2:4,5-Di-O-isopropylidene-8-tetradecyne-D-glycero-D-manno-1,2,3,4,5,6-hexol

770

770

4.60

6-Aza-5,7,12,14-tetrathiapentacene

760

806

4.60

Cyclopentasiloxane, decamethyl- (CAS)

755

775

4.60

Cyclopentasiloxane, decamethyl-

747

773

4.60

Cyclopentasiloxane, decamethyl- (CAS)

745

766

Hit Spectrum

RT

Compound Name

SI

RSI

5.31

Cyclotrisiloxane, hexamethyl- (CAS)

744

872

5.31

Cyclotrisiloxane, hexamethyl-

736

892

5.31

Cyclotrisiloxane, hexamethyl- (CAS)

723

952

5.31

6,7-Dimethoxy-2-methyl-3,4-dihydro[1-D]isoquinolinium ion

720

862

5.31

Cyclotrisiloxane, hexamethyl- (CAS)

714

815

Hit Spectrum

RT

Compound Name

SI

RSI

5.75

2,5,5-Trimethyl-2-(butylthio)cycloheptatriene

777

874

5.75

2-[5-(2-hydroxyethyl)-2-thienyl]-4,4-dimethyloxazoline

742

840

5.75

6,7-Dimethoxy-2-methyl-3,4-dihydro[1-D]isoquinolinium ion

734

857

5.75

2-(5-CHLORO-2-METHOXYPHENYL)PYRROLE

731

836

5.75

N-ethyl-1,3-dithioisoindoline

721

821

Hit Spectrum

RT

Compound Name

SI

RSI

6.34

Cyclotrisiloxane, hexamethyl- (CAS)

777

871

6.34

Cyclotrisiloxane, hexamethyl-

758

899

6.34

Cyclotrisiloxane, hexamethyl- (CAS)

752

954

6.34

Cyclotrisiloxane, hexamethyl- (CAS)

736

842

6.34

6,7-Dimethoxy-2-methyl-3,4-dihydro[1-D]isoquinolinium ion

709

859

Hit Spectrum

RT

Compound Name

SI

RSI

6.56

Cyclotrisiloxane, hexamethyl- (CAS)

728

875

6.56

Cyclotrisiloxane, hexamethyl-

721

892

6.56

Cyclotrisiloxane, hexamethyl- (CAS)

712

835

6.56

2-(5-CHLORO-2-METHOXYPHENYL)PYRROLE

707

844

6.56

Cyclotrisiloxane, hexamethyl- (CAS)

696

944

Hit Spectrum

RT

Compound Name

SI

RSI

8.00

2-Myristynoyl-glycinamide

563

620

8.00

Oxacyclotetradecan-2-one, 13-methyl-

562

721

8.00

Oxacyclotetradecan-2-one, 13-methyl- (CAS)

554

703

8.00

11,13-Dimethyl-12-tetradecen-1-ol acetate

546

595

8.00

BOROXIN, TRIS(2,3-DIMETHYLBUT-2-YL)-

545

576

Hit Spectrum

RT

Compound Name

SI

RSI

9.11

Urea, 1-(2-aminophenyl)-3-tert-butyl-

585

730

9.11

Pyrazine, 2,5-dimethyl- (CAS)

570

827

9.11

1,2-Dihydro-1,4-diphenylphthalazine

562

763

9.11

Pyrazine, 2,5-dimethyl- (CAS)

560

797

9.11

N-(2-(5-Methyl-3-furyl)ethyl)-2,5-pyrrolidinedione

558

844

Hit Spectrum

RT

Compound Name

SI

RSI

10.28

Cyclohexasiloxane, dodecamethyl- (CAS)

902

971

10.28

Cyclohexasiloxane, dodecamethyl- (CAS)

877

895

10.28

Cyclohexasiloxane, dodecamethyl-

861

886

10.28

bis(trimethylsilyl)-lorazepam

795

851

10.28

(5à,6à)-4,5-Epoxy-6-Methoxy-17-propyl-3á-phthalimidomorphinan

731

742

Hit Spectrum

RT

Compound Name

SI

RSI

11.37

Nonanal

792

899

11.37

Nonanal (CAS)

789

912

11.37

Nonanal (CAS)

787

897

11.37

Nonanal (CAS)

770

900

11.37

Nonanal (CAS)

769

896

Hit Spectrum

RT

Compound Name

SI

RSI

13.95

Acetic acid (CAS)

742

937

13.95

Acetic acid (CAS)

724

919

13.95

Acetic acid (CAS)

718

909

13.95

Acetic acid (CAS)

718

907

13.95

Acetic acid (CAS)

713

907

Hit Spectrum

RT

Compound Name

SI

RSI

16.10

5,6-Dihydro-2,4-dimethylbenz[f]isoquinoline

773

847

16.10

PHENYLPENTAMETHYLDISILOXANE

694

777

16.10

(S*,S*)-2-Hydroxy(4-methoxy-2-trimethylsilylphenyl)methyl-1-cycloheptanone

693

841

16.10

Benzene, 1-(dimethoxymethyl)-4-(1-methoxy-1-methylethyl)-

686

761

16.10

4-nitrophthalamide

676

713

Hit Spectrum

RT

Compound Name

SI

RSI

16.99

Cycloheptasiloxane, tetradecamethyl-

761

789

16.99

TETRADECAMETHYLCYCLOHEPTASILOXANE

737

757

16.99

Hexasiloxane, 1,1,3,3,5,5,7,7,9,9,11,11-dodecamethyl-

704

756

16.99

1,1,3,3,5,5,7,7,9,9,11,11-DODECAMETHYL-HEXASILOXANE

703

747

16.99

1,1,3,3,5,5,7,7,9,9,11,11-dodecamethylhexasiloxane

703

747

Hit Spectrum

RT

Compound Name

SI

RSI

17.53

1-[(2-trimethylsiloxy)vinyl]-4-trimethylsiloxy-2,6-dideuteriobenzene

581

711

17.53

1-(2-trimethylsiloxyvinyl)-4-trimethylsiloxy-3,5-dideuteriobenzene

565

683

17.53

1-Nonanol (CAS)

559

875

17.53

1-Octanol (CAS)

552

816

17.53

Octane, 3-chloro-

552

863

Hit Spectrum

RT

Compound Name

SI

RSI

19.24

9H-pyrrolo[3',4':3,4]pyrrolo[2,1-a]phthalazine-9,11(10H)-dione,10-ethyl-8-phenyl

638

748

19.24

3-(4'-Methoxyphenyl)-1-acetyl-2-phenylindolizine

617

703

19.24

7,8-Bis(trimethylsilyl)benzo(5,6-g)-1H,3H-quinazoline-2,4-dione

598

640

19.24

2,3-DICHLORO-4-(3,5-DICHLORO-2-METHOXYPHENYL)-4-OXOBUT-2-ENAMIDE

592

658

19.24

3-cyano-5,6,7-trimethoxy-2-methylthio-1-phenylindole

577

644

Hit Spectrum

RT

Compound Name

SI

RSI

22.27

QUERCETIN 7,3',4'-TRIMETHOXY

586

600

22.27

(3aS,9aS,9bR)-6,6,9aa-trimethyl-cis-perhydronaphtho[2,1-b]furan

579

771

22.27

Decane, 5,6-bis(2,2-dimethylpropylidene)-, (E,Z)- (CAS)

565

650

22.27

Decane, 5,6-bis(2,2-dimethylpropylidene)-, (E,Z)-

559

655

22.27

5,9-Methanobenzocycloocten-1(2H)-one, 3,4,5,6,7,8,9,10-octahydro-3,5-dihydroxy-3,3,7,7,9-pentamethyl-

556

669

Hit Spectrum

RT

Compound Name

SI

RSI

23.49

Cyclooctasiloxane, hexadecamethyl-

825

847

23.49

HEXADECAMETHYLCYCLOOCTASILOXANE

788

802

23.49

TETRACOSAMETHYLCYCLODODECASILOXANE

718

858

23.49

HEXADECAMETHYLCYCLOOCTASILOXANE

665

876

23.49

1,1,3,3,5,5,7,7,9,9,11,11,13,13,15,15-HEXADECAMETHYL-OCTASILOXANE

661

673

Hit Spectrum

RT

Compound Name

SI

RSI

25.75

METHOXY, PHENYL- ,OXIME

721

836

25.75

Oxime-, methoxy-phenyl-_

717

837

25.75

4-Ethylbenzoic acid, cyclopentyl ester

634

717

25.75

4-Ethylbenzoic acid, 2-butyl ester

628

684

25.75

4-Ethylbenzoic acid, cyclohexyl ester

625

698

Hit Spectrum

RT

Compound Name

SI

RSI

29.38

Cyclononasiloxane, octadecamethyl-

759

820

29.38

OCTADECAMETHYLCYCLONONASILOXANE

741

790

29.38

Tetracosamethyl-cyclododecasiloxane

680

828

29.38

1,1,1,5,7,7,7-Heptamethyl-3,3-bis(trimethylsiloxy)tetrasiloxane

641

853

29.38

1,1,3,3,5,5,7,7,9,9,11,11,13,13,15,15-HEXADECAMETHYL-OCTASILOXANE

633

668

Hit Spectrum

RT

Compound Name

SI

RSI

30.13

3-(2,2-dimethylpropylidene)bicyclo[3.3.1]nonane-2,4-dione

896

927

30.13

2,3-dicyano-7,7-dimethyl-5,6-benzonorbornadiene

894

927

30.13

Methyl 2-(3',3'-dimethyl-1'-butyn-1'-yl)-1-cyclohexenecarboxylate

888

919

30.13

5,7-dimethoxy-2,2-dimethyl-2H-chromene

887

918

30.13

methyl 4,4,7-trimethyl-4,7-dihydroindan-6-carboxylate

886

917

Hit Spectrum

RT

Compound Name

SI

RSI

36.34

2,6-di(t-butyl)-4-hydroxy-4-methyl-2,5-cyclohexadien-1-one

774

888

36.34

7-Methoxy-2,2,4,8-tetramethyltricyclo[5.3.1.0(4,11)]undecane

658

741

36.34

1-Ethyl-2,2,4a,7,7-pentamethyl-1,2,3,4,4a,5,6,7-octahydro[1,8]naphthyridine

628

668

36.34

10-Ethyl-3,3,6,9,9-pentamethyl-2,10-diazabicyclo[4..0]-1-decene

628

668

36.34

6,7-Dimethoxy-2,2-dimethyl-4-chromanone

594

624

Hit Spectrum

RT

Compound Name

SI

RSI

45.94

Benzoic acid, silver(1+) salt (CAS)

710

884

45.94

TETRAMETHYL 1-BENZOYLOXY-4,5-DIHYDROPYRIDO(1,2-A)AZEPINE-2,3,4,5-TETRACARBOXYLATE

700

921

45.94

Benzoic acid (CAS)

695

894

45.94

Benzoic acid (CAS)

692

881

45.94

Benzoic acid (CAS)

687

828

Hit Spectrum
