# Supplementary material for: Chemical and molecular characterization of metabolites from Flavobacterium sp
Source: PLoS One. 2018 Oct 17;13(10):e0205817. doi: 10.1371/journal.pone.0205817 (PMC6192653; doi:10.1371/journal.pone.0205817)
Supplement: S1 Supporting Information — (ZIP) [file pone.0205817.s002.zip › Data Set/gc-ms/EHDOSYA 2.doc]

**QUAN PEAK RESULT**

Data File:

174000040701

Sample Name:

Methanol +BHT

Acquisition Date:

05/09/17 11:50:31 AM

Instrument Method:

C:\Xcalibur\EH2017\17400040701 02\Aroma.meth

Current Processing Method:

C:\Xcalibur\EH2017\17400040701 02\process

Current Data Path:

C:\Xcalibur\EH2017\17400040701 02

RT

Peak Area

Area %

2.49

3315804

0.18

2.72

6731300

0.37

3.88

3876577

0.21

4.75

9276666

0.51

6.34

35864563

1.97

6.54

6656277

0.37

7.24

10756628

0.59

10.31

50584881

2.78

10.80

9780636

0.54

11.21

6186087

0.34

13.61

5343102

0.29

16.10

5501429

0.30

16.98

73508831

4.04

23.48

16460872

0.91

29.36

7202132

0.40

30.16

1499041014

82.46

34.61

4661718

0.26

39.55

5808981

0.32

41.55

9185608

0.51

43.67

2654326

0.15

44.20

5887829

0.32

45.80

34767631

1.91

48.58

4739482

0.26

RT

Compound Name

SI

RSI

2.49

(S)-(E)-(-)-4-Acetoxy-1-phenyl-2-dodecen-1-one

720

960

2.49

N-ethyl-1,3-dithioisoindoline

704

827

2.49

Cyclotrisiloxane, hexamethyl- (CAS)

699

841

2.49

Cyclotrisiloxane, hexamethyl- (CAS)

697

787

2.49

Cyclotrisiloxane, hexamethyl-

695

838

Hit Spectrum

RT

Compound Name

SI

RSI

2.72

Silicic acid, diethyl bis(trimethylsilyl) ester

702

782

2.72

1,4-diphenylbut-3-ene-2-ol

663

734

2.72

Trisiloxane, 1,1,3,3,5,5-hexamethyl- (CAS)

643

854

2.72

2,5,5-Trimethyl-2-(butylthio)cycloheptatriene

610

671

2.72

3,3-Diisopropoxy-1,1,1,5,5,5-hexamethyltrisiloxane

609

761

Hit Spectrum

RT

Compound Name

SI

RSI

3.88

1-[2-(nonadeuterio)trimethylsiloxyvinyl]-4-(nonadeuteriotrimethylsiloxy)-benzene

800

813

3.88

Benzoic acid, 2,4-bis(trimethylsiloxy)-, methyl ester

614

634

3.88

Benzoic acid, 2,4-bis(trimethylsiloxy)-, methyl ester (CAS)

610

626

3.88

3',4'-Dihydrospiro[acridane-9,1'(2'H)-naphthalene]

608

679

3.88

VANILETHANEDIOL 3TMS

599

599

Hit Spectrum

RT

Compound Name

SI

RSI

4.75

Cyclopentasiloxane, decamethyl- (CAS)

809

829

4.75

Cyclopentasiloxane, decamethyl-

801

822

4.75

Cyclopentasiloxane, decamethyl- (CAS)

784

893

4.75

Cyclopentasiloxane, decamethyl- (CAS)

783

801

4.75

6-Aza-5,7,12,14-tetrathiapentacene

761

804

Hit Spectrum

RT

Compound Name

SI

RSI

6.34

Cyclotrisiloxane, hexamethyl- (CAS)

779

874

6.34

Cyclotrisiloxane, hexamethyl- (CAS)

753

933

6.34

Cyclotrisiloxane, hexamethyl-

745

884

6.34

Cyclotrisiloxane, hexamethyl- (CAS)

743

843

6.34

Silicic acid, diethyl bis(trimethylsilyl) ester

730

805

Hit Spectrum

RT

Compound Name

SI

RSI

6.54

Cyclotrisiloxane, hexamethyl- (CAS)

800

874

6.54

Cyclotrisiloxane, hexamethyl- (CAS)

784

937

6.54

Cyclotrisiloxane, hexamethyl-

764

877

6.54

Cyclotrisiloxane, hexamethyl- (CAS)

761

854

6.54

2-(5-CHLORO-2-METHOXYPHENYL)PYRROLE

734

845

Hit Spectrum

RT

Compound Name

SI

RSI

7.24

1,1,3,3,5,5,7,7,9,9,11,11,13,13-tetradecamethylheptasiloxane

672

894

7.24

Silane, dimethoxymethyl-

649

683

7.24

3,5-Dithiahexanol 5,5-dioxide

648

728

7.24

3,5-Dithiahexanol 5,5-dioxide

646

726

7.24

Silane, dimethoxymethyl- (CAS)

642

675

Hit Spectrum

RT

Compound Name

SI

RSI

10.31

Cyclohexasiloxane, dodecamethyl- (CAS)

909

972

10.31

Cyclohexasiloxane, dodecamethyl- (CAS)

885

900

10.31

Cyclohexasiloxane, dodecamethyl-

867

887

10.31

bis(trimethylsilyl)-lorazepam

806

868

10.31

Cyclohexasiloxane, dodecamethyl- (CAS)

728

874

Hit Spectrum

RT

Compound Name

SI

RSI

10.80

Lupulon

581

607

10.80

Tungsten, tris(ã-allyl)(ã-1,3-dimethylallyl)-

581

635

10.80

6-Chloro-6-cyano-4,5-diphenyl-3-ethoxy-2-methyl-2,4-cyclohexadienone

581

868

10.80

WOLFRAM, TRIS(PI-ALLYL)(1,3-DIMETHYLALLYL)-

581

635

10.80

EUPULONE

579

605

Hit Spectrum

RT

Compound Name

SI

RSI

11.21

Benzyloxy(butyl)dimethylsilane

605

666

11.21

Allyl(dimethyl)benzyloxysilane

586

655

11.21

1-Methyl-2-dimethyl-(allyl)-silyloxybenzene

583

664

11.21

SILANE, (1,1-DIMETHYLETHYL)DIMETHYL(PHENYLMETHOXY)-

576

733

11.21

Benzyloxydimethyloctylsilane

564

639

Hit Spectrum

RT

Compound Name

SI

RSI

13.61

1,4-diphenylbut-3-ene-2-ol

686

774

13.61

2,5,5-Trimethyl-2-(butylthio)cycloheptatriene

650

727

13.61

Silicic acid, diethyl bis(trimethylsilyl) ester

636

748

13.61

N-ethyl-1,3-dithioisoindoline

635

790

13.61

Cyclotrisiloxane, hexamethyl- (CAS)

634

779

Hit Spectrum

RT

Compound Name

SI

RSI

16.10

5,6-Dihydro-2,4-dimethylbenz[f]isoquinoline

686

820

16.10

4-nitrophthalamide

682

750

16.10

Benzene, 1-(dimethoxymethyl)-4-(1-methoxy-1-methylethyl)-

661

762

16.10

Acetic acid, N'-[3-(1-hydroxy-1-phenylethyl)phenyl]hydrazide

654

673

16.10

ACETIC ACID N'-[2-(1-HYDROXY-1-PHENYL-ETHYL)-PHENYL]-HYDRAZIDE

649

668

Hit Spectrum

RT

Compound Name

SI

RSI

16.98

Cycloheptasiloxane, tetradecamethyl-

772

795

16.98

TETRADECAMETHYLCYCLOHEPTASILOXANE

746

759

16.98

1,1,3,3,5,5,7,7,9,9,11,11-DODECAMETHYL-HEXASILOXANE

704

751

16.98

1,1,3,3,5,5,7,7,9,9,11,11-dodecamethylhexasiloxane

704

751

16.98

Hexasiloxane, 1,1,3,3,5,5,7,7,9,9,11,11-dodecamethyl-

699

749

Hit Spectrum

RT

Compound Name

SI

RSI

23.48

Cyclooctasiloxane, hexadecamethyl-

821

856

23.48

HEXADECAMETHYLCYCLOOCTASILOXANE

780

804

23.48

TETRACOSAMETHYLCYCLODODECASILOXANE

714

868

23.48

HEXADECAMETHYLCYCLOOCTASILOXANE

660

881

23.48

Benzeneacetic acid, à,3,4-tris[(trimethylsilyl)oxy]-, methyl ester (CAS)

647

737

Hit Spectrum

RT

Compound Name

SI

RSI

29.36

Cyclononasiloxane, octadecamethyl-

799

848

29.36

OCTADECAMETHYLCYCLONONASILOXANE

773

813

29.36

Tetracosamethyl-cyclododecasiloxane

710

854

29.36

1,1,3,3,5,5,7,7,9,9,11,11,13,13,15,15-HEXADECAMETHYL-OCTASILOXANE

687

718

29.36

Octasiloxane, 1,1,3,3,5,5,7,7,9,9,11,11,13,13,15,15-hexadecamethyl-

676

712

Hit Spectrum

RT

Compound Name

SI

RSI

30.16

Benzene, 1,1'-(3-methyl-1,3-butadienylidene)bis- (CAS)

949

955

30.16

3-(2,2-dimethylpropylidene)bicyclo[3.3.1]nonane-2,4-dione

944

949

30.16

2,3-dicyano-7,7-dimethyl-5,6-benzonorbornadiene

943

948

30.16

methyl 4,4,7-trimethyl-4,7-dihydroindan-6-carboxylate

938

943

30.16

Phenol, 2,6-bis(1,1-dimethylethyl)-4-methyl- (CAS)

936

937

Hit Spectrum

RT

Compound Name

SI

RSI

34.61

1,1,3,3,5,5,7,7,9,9,11,11,13,13,15,15-HEXADECAMETHYL-OCTASILOXANE

748

775

34.61

Octasiloxane, 1,1,3,3,5,5,7,7,9,9,11,11,13,13,15,15-hexadecamethyl-

743

777

34.61

Cyclononasiloxane, octadecamethyl-

714

751

34.61

1,1,3,3,5,5,7,7,9,9,11,11,13,13-TETRADECAMETHYL-HEPTASILOXANE

706

742

34.61

1,1,3,3,5,5,7,7,9,9,11,11,13,13-tetradecamethylheptasiloxane

706

742

Hit Spectrum

RT

Compound Name

SI

RSI

39.55

1,1,3,3,5,5,7,7,9,9,11,11,13,13,15,15-HEXADECAMETHYL-OCTASILOXANE

712

740

39.55

TETRACOSAMETHYLCYCLODODECASILOXANE

710

927

39.55

Octasiloxane, 1,1,3,3,5,5,7,7,9,9,11,11,13,13,15,15-hexadecamethyl-

708

744

39.55

Tetracosamethyl-cyclododecasiloxane

701

835

39.55

Cyclooctasiloxane, hexadecamethyl-

699

774

Hit Spectrum

RT

Compound Name

SI

RSI

41.55

Benzoic acid, silver(1+) salt (CAS)

741

893

41.55

Benzoic acid (CAS)

739

854

41.55

TETRAMETHYL 1-BENZOYLOXY-4,5-DIHYDROPYRIDO(1,2-A)AZEPINE-2,3,4,5-TETRACARBOXYLATE

731

929

41.55

Benzoic acid (CAS)

728

838

41.55

Benzoic acid (CAS)

726

854

Hit Spectrum

RT

Compound Name

SI

RSI

43.67

5H-Dibenzo[a,d]cyclohepten-5-one, 10,11-dihydro-

724

790

43.67

1,2-Diphenylethylene

718

906

43.67

Benzene, 1,1'-(1,2-ethenediyl)bis-, (E)- (CAS)

711

852

43.67

Anthracene, 9,10-dihydro-

708

857

43.67

(E)-Stilbene

707

854

Hit Spectrum

RT

Compound Name

SI

RSI

44.20

Cyclononasiloxane, octadecamethyl-

734

790

44.20

Tetracosamethyl-cyclododecasiloxane

733

877

44.20

OCTADECAMETHYLCYCLONONASILOXANE

720

761

44.20

Iron, monocarbonyl-(1,3-butadiene-1,4-dicarbonic acid, diethyl ester) a,a'-dipyridyl

692

720

44.20

SILIKONFETT SE30 (GREVELS)

692

720

Hit Spectrum

RT

Compound Name

SI

RSI

45.80

Methyl 2-{4'-[(E)-2"-Nitroethenyl]phenyloxy}ethyl pentanedioate

660

911

45.80

Decanoic acid, decyl ester

631

644

45.80

Decanoic acid, decyl ester (CAS)

630

644

45.80

(s)-n-(2',2'-dimethylpropyliden)methionin-(n-methylamid)

624

629

45.80

Butanamide, N-methyl-4-(methylthio)-2-(2,2-dimethylpropylidene)amino-

621

628

Hit Spectrum

RT

Compound Name

SI

RSI

48.58

Cyclononasiloxane, octadecamethyl-

723

780

48.58

1,1,3,3,5,5,7,7,9,9,11,11,13,13,15,15-HEXADECAMETHYL-OCTASILOXANE

718

749

48.58

Tetracosamethyl-cyclododecasiloxane

717

874

48.58

Octasiloxane, 1,1,3,3,5,5,7,7,9,9,11,11,13,13,15,15-hexadecamethyl-

711

754

48.58

OCTADECAMETHYLCYCLONONASILOXANE

704

742

Hit Spectrum
